# On-chip deterministic arbitrary-phase-controlling

**DOI:** 10.1515/nanoph-2025-0132

**Published:** 2025-07-02

**Authors:** Rui Ma, Chu Li, Qiuchen Yan, Xinyi Wang, Ruiqi Wang, Yufei Wang, Yumeng Chen, Yan Li, Cuicui Lu, Jianwei Wang, Xiaoyong Hu, Che Ting Chan, Qihuang Gong

**Affiliations:** State Key Laboratory for Mesoscopic Physics & Department of Physics, Collaborative Innovation Center of Quantum Matter & Frontiers Science Center for Nano-Optoelectronics, Peking University, Beijing 100871, P.R. China; Peking University Yangtze Delta Institute of Optoelectronics, Nantong, Jiangsu 226010, P.R. China; Collaborative Innovation Center of Extreme Optics, Shanxi University, Taiyuan, Shanxi 030006, P.R. China; Hefei National Laboratory, Hefei 230088, P.R. China; Laboratory of Advanced Optoelectronic Quantum Architecture and Measurements of Ministry of Education, Beijing Key Laboratory of Nanophotonics and Ultrafine Optoelectronic Systems, Center for State Key Laboratory of Chips and Systems for Advanced Light Field Display, Interdisciplinary Science of Optical Quantum and NEMS Integration, School of Physics, Beijing Institute of Technology, Beijing 100081, P.R. China; Department of Physics and Institute for Advanced Study, The Hong Kong University of Science and Technology, Clear Water Bay, Kowloon, Hong Kong, P.R. China

**Keywords:** deterministic arbitrary-phase-controlling, optical permutation circuit, optical chip, three-waveguide configuration

## Abstract

The stable on-chip deterministic arbitrary-phase-controlling of signal light in micro/nanometer spatial scale is an extremely important basis for large-scale and high-density integrated photonic information processing chips. Conventional phase-controlling methods face with serious limitation of unavoidable crosstalk, length distortion, and fabrication error. To date, it is still a great challenge to achieve deterministic and wide-range on-chip arbitrary-phase-controlling. Here, we report an effective strategy of three-waveguide coupled configuration to realize on-chip deterministic arbitrary-phase-controlling (ranging from 0 to 2*π*) by combing the dynamic phase and the geometric phase. Based on this strategy, quantum gate operations in an optical permutation-group circuit are successfully realized in femtosecond-laser direct writing sample. To extend the feasibility of this method, on-chip silicon-based deterministic arbitrary-phase-controlling in the optical communication range is also experimentally verified. Our work not only paves the way for fundamental research in chip-scale novel optical devices but also promotes the study of topological quantum computing.

## 1 Introduction

The large-scale and high-density integration of nanophotonic devices is one of the essential bases of ultracompact integrated photonic chips, which have great potential application in ultrahigh-speed and ultrabroad-band information processing [1], [2], [3], [4], [5], [6], [7], [8], [9], [10]. The deterministic arbitrary-phase-controller with a small footprint is a vital step for the realization of large-scale and high-density integration. Conventional on-chip phase-controlling methods predominantly rely on electro-optic or thermo-optic controlling approaches [11], [12], [13], [14], [15], [16], [17], [18], [19], exemplified by configurations such as the Mach–Zehnder interferometer (MZI) and micro-ring resonator (MRR) [20], [21], [22], [23], [24]. While these methods enable arbitrary-phase-controlling, the precision and feasibility are deteriorated by unavoidable crosstalk, length distortion, and fabrication errors in waveguides. Moreover, phase controlling at shorter wavelengths is more sensitive to the alterations and defects above [25], [26], [27], [28], [29], [30]. Since the temperature-induced refractive index change of the target waveguides is mainly achieved by applying voltage over the controller electrodes, it influences the refractive index of surrounding waveguides at the same time, deteriorating the phase controlling ability of nearby MZIs. Such effect is even more serious on more integrated chips where the gaps between unit devices shrink. This not only limits the integration scale of the integrated photonic chip to a great extent but also brings great difficulties to the debugging process. When considering the computing capacity, existing on-chip calculation based on MZI or MRR only executes the addition and the multiplication operations that belong to the Abelian group but has difficulties in non-Abelian group operation that is more promising in sophisticated computing. For example, the permutation operation is a classic non-Abelian operation, and non-Abelian braiding can be achieved through the cascade of permutations, which is an important information processing operation in topological quantum computing. It is still a great challenge to achieve the deterministic arbitrary-phase-controllers with a small footprint for the non-Abelian operations, which are very important for future information processing.

Here, we propose an effective strategy, three-waveguide coupled system, to realize on-chip deterministic arbitrary-phase-controlling, which has the enhanced resistance against fabrication errors and is able to achieve large-scale integration and the quantum gate operations in optical permutation-group circuits. The three waveguides are arranged in the configuration of an isosceles triangle to realize the deterministic arbitrary-phase-controlling based on the dynamic phase and the geometric phase. The dynamic phase accumulates from the eigen-energetics and travel time of the intervening state evolution. The geometric phase is usually determined solely by the geometry of the traversed loop [31]. In optics, the phases accumulated during the evolution process of Hamiltonian is also considered to be the geometric phase [32]. The dynamic phase is introduced in the configurations when the light travels along waveguides and the geometric phase is introduced when the light is coupled between waveguides. Geometric phase is considered to be stable, capable of resisting sample fabrication errors, such as the non-Abelian braiding configurations based on the Berry phase [33], [34], [35], [36], [37], [38], [39], [40], [41]. As for the waveguide system, the geometric phase controlling ranges only single value 0 or *π* [32], [42], [43]. We compare the phase controlling schemes as shown in Table 1.

**Table 1:** The comparison of different phase controlling methods.

Citation	Phase controlling method	Configuration	Application platform	Phase controlling range	Limited by fabrication error
[17], [18]	Dynamic phase	Mach–Zehnder interferometer	Femtosecond laser direct writing	0–2*π*	Yes
[25], [26], [28], [29]		Mach–Zehnder interferometer	Silicon-based	0–2*π*	Yes
[32]	Geometric phase	Waveguides	Femtosecond laser direct writing	Only 0, *π*	No
[43]		Two identical dual-waveguide	Silicon-based	Only 0, *π*	No
This work	Dynamic phase + geometric phase	Three-waveguide	Femtosecond laser direct writing or silicon-based	0–2*π*	No

The proposed deterministic phase-controller based on three waveguides can change the phase by adjusting the coupling parameters of the waveguides, significantly reducing the crosstalk problem of traditional thermo-optic controlling from origin. Our phase-controller has lower energy consumption and higher computing power because no external electrodes is contained. The phase controlling ranging from 0 to 2*π* can be achieved, and our measured results of femtosecond-laser direct writing samples and the on-chip silicon-based samples confirm the feasibility of this strategy. Quantum gate operations in optical permutation-group circuit are successfully realized, maintaining its outstanding ability of stability in quantum state. Our work not only paves the way for fundamental research in chip-scale novel optical devices but also advances the study of topological quantum computing.

## 2 Results

### 2.1 Theory of on-chip deterministic arbitrary-phase-controller

**Figure 1:**
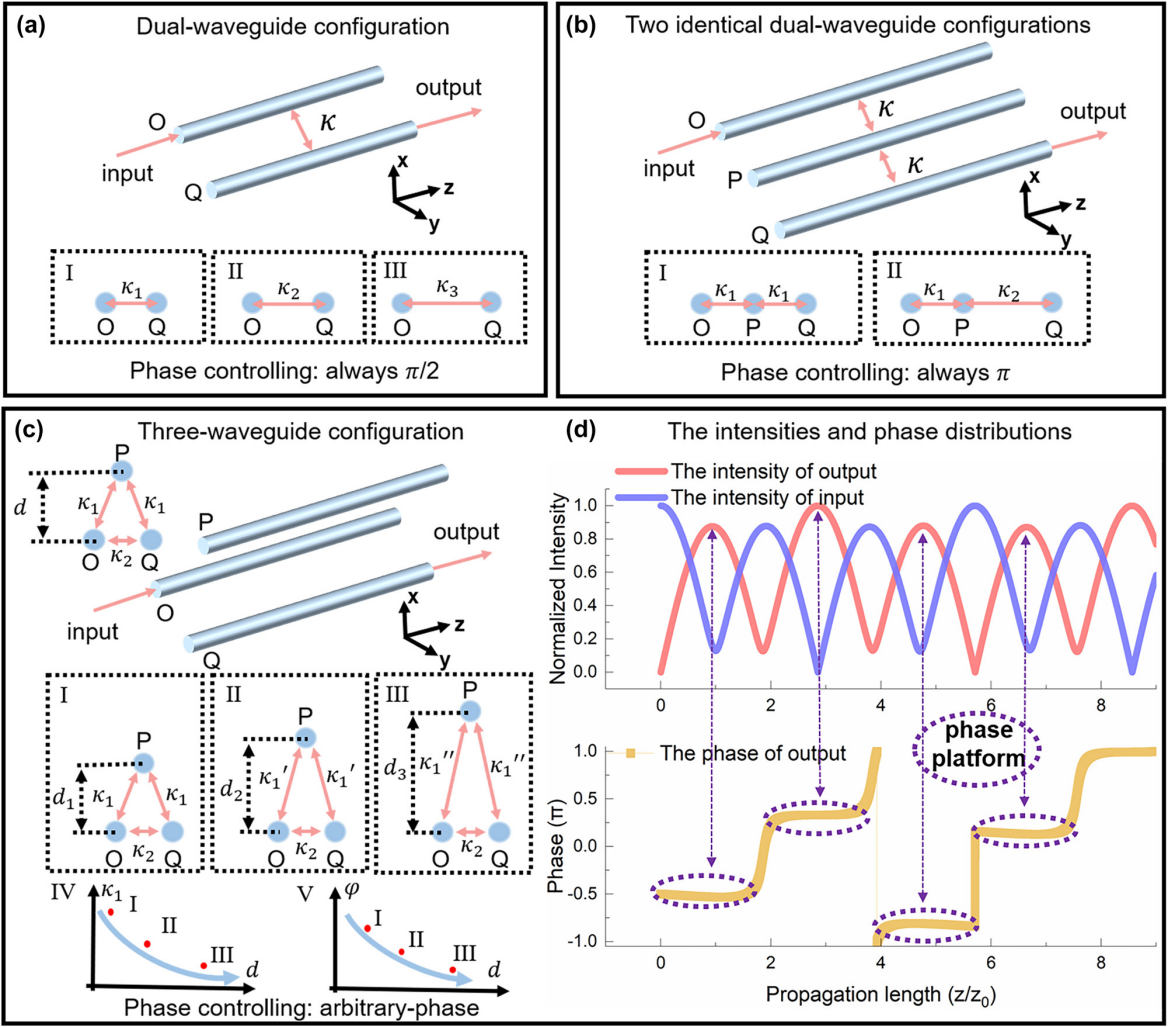
The Schematic diagram of waveguide coupling system. (a) The dual-waveguide configuration composed of two identical waveguides. I, II, III show that the coupling coefficient changes with the distance between waveguides. No matter what the coupling coefficient is, the dual-waveguide configuration can only realize *π*/2 phase controlling; (b) the two identical dual-waveguide configurations. For the coupling shown by I and II, the two identical dual-waveguide configurations can only realize *π* phase controlling; (c) the three-waveguide configuration, the structure parameter *d* represent the distance between the waveguide P and the plane determined by the waveguide O and waveguide Q. The coupling coefficient *κ*_1_ decreases with the increase of *d*, then the arbitrary-phase-controlling can be achieved; (d) the optical field intensities in waveguide O, waveguide Q, and the phase distributions in waveguide Q of the three-waveguide configuration, which is obtained by solving the Schrodinger equation in the waveguide configuration and angling the function. Corresponding to each coupling, the phase platforms are obvious.

Consider a dual-waveguide configuration composed of two identical waveguides denoted as O and Q, as illustrated in Figure 1(a). The transmission of the optical field within them follows the form of the Schrödinger-like equation, i.e., $H\left(z\right)\left|\psi \left(z\right)\right\rangle =-i\frac{\partial }{\partial z}\left|\psi \left(z\right)\right\rangle$ . The Hamiltonian can be then expressed as $H=\left(\begin{array}{cc} \beta & \kappa \\ \kappa & \beta \end{array}\right)$ , where *β* is the propagation constants of the waveguide, and *κ* is the coupling coefficient between the two waveguides, which changes with the distance between waveguides. Since the two waveguides are identical, setting *β =* 0 eliminates the propagation constant for a simplified analysis. In this case, the Hamiltonian becomes $H=\left(\begin{array}{cc} 0 & \kappa \\ \kappa & 0 \end{array}\right)$ . Its eigenvector takes the form $\left|\psi \left(z\right)\right\rangle =\left[\phi_{O}\left(z\right),\phi_{Q}\left(z\right)\right]$ , where $\phi_{O}\left(z\right)$ and $\phi_{Q}\left(z\right)$ represent the wave functions in the two waveguides, respectively. The eigenvalues of the system are found to be *λ* = ±*κ*, and the corresponding eigenvectors are as Equation (1) shows.

(1) $$\left\{\begin{array}{cc} \psi_{1}=\frac{1}{\sqrt{2}}{\left(1,1\right)}^{T}\; & \lambda =\kappa \\ \psi_{2}=\frac{1}{\sqrt{2}}{\left(1,-1\right)}^{T}\; & \lambda =-\kappa \end{array}\right.$$

The solutions of the Equation (1) are odd-mode and even-mode in the dual-waveguide configuration, respectively. The change of any mode, light intensity, and phase in the system can be obtained by linear superposition of this series of odd mode and even mode. If the light is incident into waveguide O at *z* = 0, the initial state vector can be written as $\varphi_{z=0}={\left(1,0\right)}^{T}$ . Expanding it in terms of the eigenvectors, the vector can be written as $\varphi_{z=0}={\left(1,0\right)}^{T}=\frac{1}{2}\left[{\left(1,1\right)}^{T}+{\left(1,-1\right)}^{T}\right]=\frac{\sqrt{2}}{2}\left(\psi_{1}+\psi_{2}\right)$ . Thus, the evolution process of the state vector in the dual-waveguide configuration can be described as Equation (2) shows:

(2) $$\begin{array}{cc} \varphi_{z} & =\frac{\sqrt{2}}{2}\left(\psi_{1}{\text{e}}^{-\text{i}\kappa z}+\psi_{2}{\text{e}}^{\text{i}\kappa z}\right)\\ & =\frac{1}{2}\left[{\left(1,1\right)}^{T}{\text{e}}^{-\text{i}\kappa z}+{\left(1,-1\right)}^{T}{\text{e}}^{\text{i}\kappa z}\right]\\ & =\frac{1}{2}\left(\begin{array}{c} {\text{e}}^{-\text{i}\kappa z}+{\text{e}}^{\text{i}\kappa z}\\ {\text{e}}^{-\text{i}\kappa z}-{\text{e}}^{\text{i}\kappa z} \end{array}\right)=\left(\begin{array}{c} \cos\left(\kappa z\right)\\ -i\text{sin}\left(\kappa z\right) \end{array}\right) \end{array}$$

When the light completely transfers from waveguide O to waveguide Q, it is easy to obtain $\kappa z=\frac{\pi }{2}$ because $\varphi_{z}=\left(\begin{array}{c} 0\\ -i \end{array}\right)=\left(\begin{array}{c} 0\\ -{\text{e}}^{-\text{i}\frac{\pi }{2}} \end{array}\right)$ . Therefore, in the evolution process of this dual-waveguide configuration, a phase shift of *π*/2 is introduced. It is observed that during the light transfer process, there is always a *π*/2 phase difference between waveguide Q and waveguide O. This phase shift is independent of the distance that the light travels in the waveguides and is referred to as geometric phase. In addition, if two identical dual-waveguide configurations are parallelly cascaded, a three-waveguide configuration is formed, as shown in Figure 1(b). We label these three waveguides as O, P, and Q, assume that there is no coupling strength between waveguides O and Q, and only consider the interactions between the O–P and P–Q pairs of dual-waveguide configurations. Based on the above analysis, if light is incident from waveguide O and eventually outputs from waveguide Q, it will acquire two geometric phases of *π*/2 in the transmission process through the O–P and P–Q pairs of dual-waveguide configurations. Consequently, the light output from waveguide Q will experience a combined geometric phase of *π* due to the superposition of two *π*/2 geometric phases.

However, if the coupling strength between the O–Q pair of the dual-waveguide configuration in the aforementioned three-waveguide system is taken into consideration, the accumulated phase will change. The schematic of this three-waveguide system is illustrated in Figure 1(c). For ease of calculation, we assume that the coupling coefficients within the O–P and P–Q pairs of dual-waveguide configurations are denoted as *κ*_1_, and the coupling coefficient within the O–Q dual-waveguide configuration is *κ*_2_. Similar to the case of two waveguides, the propagation constants *β* for the three waveguides are also set to be zero for simplicity, so that we can eliminate the dynamic phase brought by propagating along the identical three waveguides. When the light is incident from waveguide O and output from waveguide Q, the waveguide P serves as an intermediary for controlling the light. In this case, the Hamiltonian for the O–P–Q three-waveguide configuration is given by $H=\left(\begin{array}{ccc} 0 & \kappa_{1} & \kappa_{2}\\ \kappa_{1} & 0 & \kappa_{1}\\ \kappa_{2} & \kappa_{1} & 0 \end{array}\right)$ , simply obtaining the eigenvalues and eigenvectors of the system, shown in Equations (3)–(5).

(3)ψ1=−1,0,1Tλ1=−κ2(4)ψ2=1,−4κ12−κ22−κ28κ12+κ22κ1−3κ2+8κ12+κ22,1Tλ2=12κ2−8κ12+κ22(5)ψ3=1,−−4κ12+κ22−κ28κ12+κ22κ13κ2+8κ12+κ22,1Tλ3=12κ2+8κ12+κ22

If light is incident into waveguide O where z is equal to zero, the initial state vector can be expressed as $\varphi_{z=0}={\left(1,0,0\right)}^{T}$ . Expanding it in terms of the eigenvectors, it takes the form as *φ*_*z*=0_ = *Aψ*_1_ + *Bψ*_2_ + *Cψ*_3_, where *A*, *B*, *C* are the coefficients related to the coupling coefficient *κ*_1_ and *κ*_2_. Furthermore, the evolution process of the state vector in the three-waveguide configuration can be expressed as Equation (6) shows.

(6) $$\varphi_{z}=A\psi_{1}{\text{e}}^{-\text{i}\lambda_{1}z}+B\psi_{2}{\text{e}}^{-\text{i}\lambda_{2}z}+C\psi_{3}{\text{e}}^{-\text{i}\lambda_{3}z}$$

For simplicity, we introduce the parameter $a=\frac{\kappa_{2}}{\kappa_{1}}$ to represent the ratio between the two different coupling strengths. By substituting *κ*_2_ = *aκ*_1_ into the previous expression, and replacing *A*, *B*, *C*, *ψ*_1_, *ψ*_2_, *ψ*_3_, the evolution function for the state vector from waveguide O in the O–P–Q three-waveguide configuration can be obtained. Since the light is incident from waveguide O and output from waveguide Q, the evolution functions of the optical fields in waveguides O and Q are particularly crucial to analyze the input and output states of light in the three-waveguide configuration. The evolution functions of the optical fields in waveguides O and Q from *φ*_*z*_ are shown in Equations (7) and (8).

(7) $$O\left(a,\kappa_{1},z\right)=\cos\left(Jz\right){\text{e}}^{\frac{1}{4}\text{i}\kappa_{1}\left(a-\sqrt{8+a^{2}}\right)z}+K{\text{e}}^{-\frac{1}{2}\text{i}a\kappa_{1}z}\cdot 2\text{sin}\left(Lz\right)\;$$

(8) $$Q\left(a,\kappa_{1},z\right)=-i\text{sin}\left(Jz\right){\text{e}}^{\frac{1}{4}\text{i}\kappa_{1}\left(a-\sqrt{8+a^{2}}\right)z}+K{\text{e}}^{-\frac{1}{2}\text{i}a\kappa_{1}z}\cdot 2\text{sin}\left(Lz\right)$$

where *z* represents the optical transmission distance in the three-waveguide configuration, and all the parameters *J*, *K*, and *L* are related to the three-waveguide configuration with $K=\frac{5a^{2}-3a\sqrt{8+a^{2}}+4}{10a^{2}+16+a^{4}-\left(10a-a^{3}\right)\sqrt{8+a^{2}}}$ , $J=\frac{3}{4}a\kappa_{1}z+\frac{1}{4}\kappa_{1}\sqrt{8+a^{2}}$ , and $L=\frac{1}{2}\kappa_{1}\sqrt{8+a^{2}}$ . The optical field intensities in waveguide O, waveguide Q, and the phase distributions in waveguide Q, which is obtained by Equation (8) in the waveguide configuration, are shown in Figure 1(d). Defining the length of the waveguide as *z*_0_ when the light first reaches the highest transmission in the O–P–Q three-waveguide configuration, both the intensity and phase distributions can be normalized. It is observed that the optical field intensity variation exhibits periodic characteristics. By choosing appropriate parameter values for *a* and *κ*_1_, it is able to achieve complete transmission of optical field intensity from waveguide O to waveguide Q, similar to the two identical dual-waveguide configurations. For instance, when the optical field intensity in waveguide O reaches its second minimum, i.e., the intensity in waveguide O approaches zero, while the intensity in waveguide Q becomes maximum, concentrating almost all the energy in this three-waveguide configuration. Furthermore, unlike the *π* phase in the two identical dual-waveguide configurations, this three-waveguide configuration can achieve arbitrary phase accumulation at the output port of waveguide Q within the range from 0 to 2*π*, as indicated by the yellow line in Figure 1(d), which is also obtained by Equation (8). Moreover, although the light intensity changes as the light is transmitted, the phase remains the same, which shows that the solved phase variation curve for waveguide Q exhibits periodic plateau characteristics. During these plateau phases, the optical field phase in waveguide Q remains constant, providing stable phase values over a certain evolution range, as explained in the subsequent Equation (9). The dynamic phase and the geometric phase are combined to produce the stable phase values here. Therefore, through the O–P–Q three-waveguide configuration, the stable phase control within a certain range of optical transmission distance *z* is achieved. This phase can be controlled arbitrarily by changing the ratio *a* between the two different coupling strengths *κ*_1_ and *κ*_2_ in a three-waveguide configuration, significantly enhancing the flexibility in manipulating the optical field.

**Figure 2:**
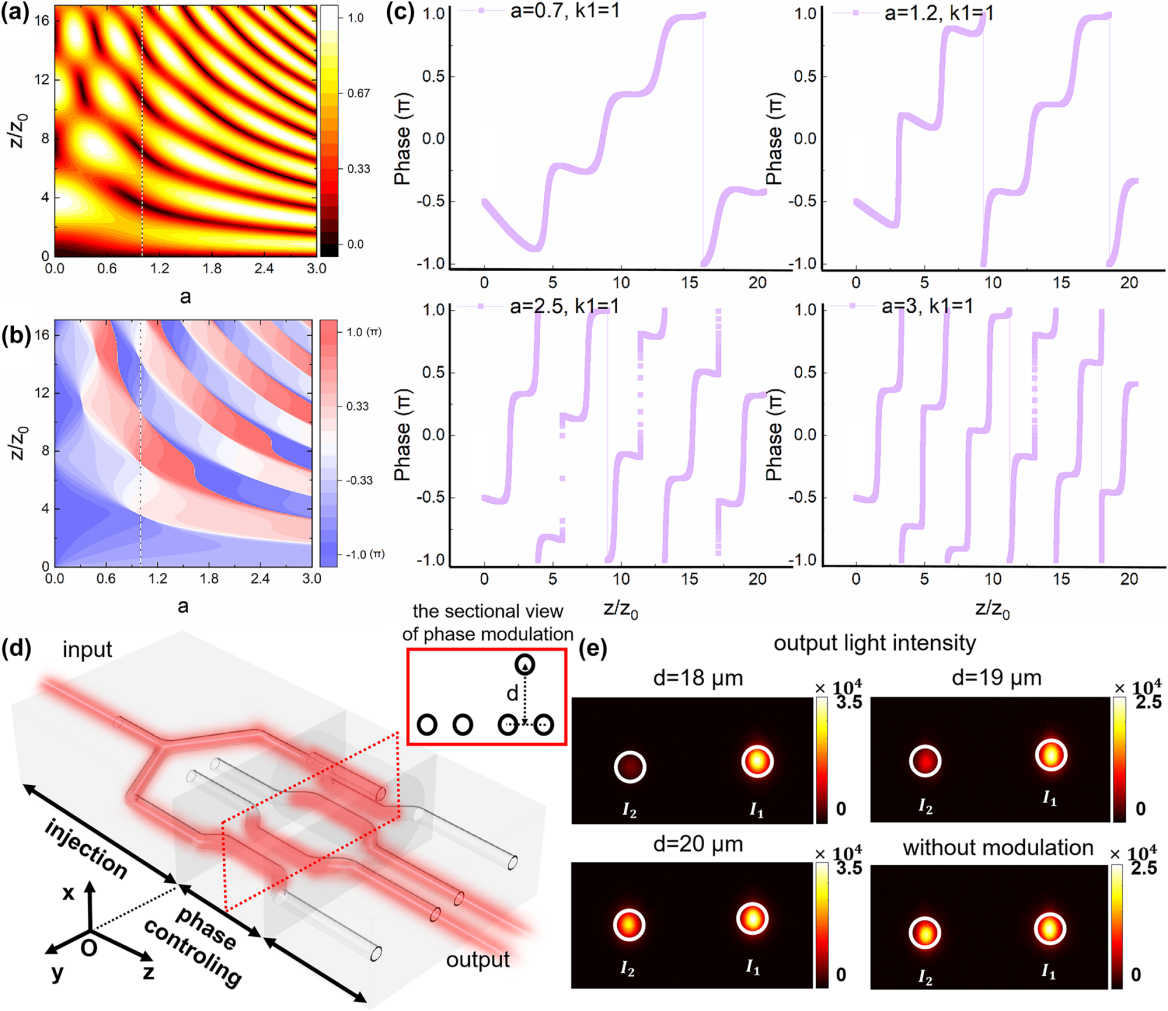
The theoretical and experimental results of the three-waveguide configuration. (a) The distribution of output optical field intensity of waveguide Q with respect to changes in the O–P–Q three-waveguide configuration when *κ*_1_ = 1, the line at *a* = 1 is the area where the singularity appears in the theory in the O–P–Q three-waveguide configuration when *a* = 1. (b) The distribution of output optical field phase of waveguide Q with respect to changes in the O–P–Q three-waveguide configuration when *κ*_1_ = 1, the line at *a* = 1 is the area where the singularity appears in the theory in the O–P–Q three-waveguide configuration when *a* = 1. (c) The different phase changes caused by varying parameters *a* when *κ*_1_ = 1; (d) the designed circuits for experimental verification of the arbitrary phase value controlling capability of the O–P–Q three-waveguide configuration. The red box shows the cross section of the phase controller, which consists of a three-waveguide configuration and a dual-waveguide configuration with the same length; (e) the output optical field intensity distribution in experiments for the O–P–Q three-waveguide configuration.

Changing the values of *a* and *κ*_1_ will alter the distribution of output optical field intensity at different positions in the O–P–Q three-waveguide configuration. Figure 2(a) illustrates the variation of optical field intensity in waveguide Q with respect to changes in *a* when *κ*_1_ = 1. It is noteworthy that the phase variation also exhibits periodic characteristics, and its periodicity is consistent with that of the optical field intensity. Changing the values of *a* and *κ*_1_ will similarly affect the phase output. Figure 2(b) and (c) demonstrates different phase changes caused by varying parameters *a*. As the coupling ratio *a* increases, the phase value controlling capability of the O–P–Q three-waveguide configuration gradually enhances, resulting in a larger phase value shift. The phase shift is caused by the hopping of light between different waveguides. Since a phase shift occurs during each hopping, it is represented in Figure 2(c) as a series of discontinuous phase plates. When the light in the waveguide is fully transmission, the corresponding mode is not an eigenstate of the system, but a linear superposition of a series of eigenstates. This multi-eigenstate effect will enable arbitrary controlling of different phases. However, the theoretical calculation results show that the coupling coefficient *κ*_1_ does not affect the phase value controlling capability, and it only influences the periodicity of phase and optical field intensity variations in the three-waveguide configuration. This indicates that the O–P–Q three-waveguide configuration possesses the capability for arbitrary-phase-controlling. We can then derive the relationship between phase value and the three-waveguide configuration parameter and the optical transmission distance in the three-waveguide configuration *z*, seen in Equation (9).

(9) $$\varphi =\arctan\left\{-\frac{\frac{1}{2}\sin\left(a\kappa_{1}z\right)+K\text{sin}\left[Nz\right]+M\text{sin}\left[Nz\right]}{-\frac{1}{2}\cos\left(a\kappa_{1}z\right)+K\text{cos}\left[Nz\right]+M\text{cos}\left[Nz\right]}\right\}$$

where all the parameters *K*, *M*, and *N* are related to the three-waveguide configuration parameters *a*, *κ*_1_, $K=\frac{5a^{2}-3a\sqrt{8+a^{2}}+4}{10a^{2}+16+a^{4}-\left(10a-a^{3}\right)\sqrt{8+a^{2}}}$ , $M=\frac{5a^{2}+3a\sqrt{8+a^{2}}+4}{10a^{2}+16+a^{4}+\left(10a-a^{3}\right)\sqrt{8+a^{2}}}$ , and $N=\frac{1}{2}\left(a-\sqrt{8+a^{2}}\right)\kappa_{1}$ .

Equation (9) represents the analytical expression of the phase controlling capability of the O–P–Q three-waveguide configuration. We designed a dual-arm interferometer to test the phase shifting capability of our device, as shown in Figure 2(d), where one arm is a three-waveguide configuration and the other arm is a dual-waveguide configuration, and the phase difference between the two arms is experimentally measured, which is obtained by the interference experiment measurements from the output optical field at the end of the circuit. By utilizing the O–P–Q three-waveguide configuration and an O–Q dual-waveguide configuration of equal length for interference, where the geometric phase in the O–Q dual-waveguide configuration remains fixed at *π*/2 and does not vary, the phase differences between the O–P–Q three-waveguide configuration and the O–Q dual-waveguide configuration can be measured, allowing to determine the controlling capability of waveguide P on the O–P–Q three-waveguide configuration. The phase analyses and comparisons between the dual-waveguide configuration and the three-waveguide configuration are discussed in the Supplementary Information II.

Assuming that the phase difference between the O–P–Q three-waveguide configuration and the O–Q configuration is *φ*, when the light beam is split and incident into both configurations, the light output from waveguide Q in the O–P–Q configuration and the light output from waveguide O in the O–Q configuration together form a set of states represented by $\frac{1}{\sqrt{2}}\left(\begin{array}{c} {\text{e}}^{\text{i}\varphi }\\ 1 \end{array}\right)$ . Through a 1:1 directional coupler, this set of states can be described by the matrix $U=\frac{1}{\sqrt{2}}\left(\begin{array}{cc} 1 & i\\ i & 1 \end{array}\right)$ , and the output state is given by $\psi =\frac{1}{\sqrt{2}}U\left(\begin{array}{c} {\text{e}}^{\text{i}\varphi }\\ 1 \end{array}\right)$ . Therefore, the calculated optical field intensities from the two output ports of the directional coupler are $I_{1}=\frac{1}{2}I_{0}\left(1+\sin\varphi \right)$ and $I_{2}=\frac{1}{2}I_{0}\left(1-\sin\varphi \right)$ . By measuring the ratio of *I*_1_ to *I*_2_, i.e., $\eta =\frac{I_{1}}{I_{2}}=\frac{1+\sin\varphi }{1-\sin\varphi }$ , the phase shift *φ* can be determined. Using the laser direct writing waveguide platform for experiments, the output optical field intensity distribution is obtained and shown in Figure 2(e). Detailed results can be found in Table 2:

**Table 2:** The output optical field intensity distribution and the phase controlling for O–P–Q three-waveguide configuration.

*d*/μm	Theory *ϕ* (^*^ *π*)	The coupling length = 6 mm			The coupling length = 5.8 mm		
		*I*_1_	*I*_2_	Phase *ϕ* (^*^ *π*)	*I*_1_	*I*_2_	Phase *ϕ* (^*^ *π*)
∞	0	3.6	3.4	0	3.1	3.0	0
18	0.25	5.1	0.7	0.27	5.8	1.0	0.25
19	0.14	4	1.6	0.14	3.5	1.5	0.13
20	0.05	3.1	2.4	0.04	3.0	2.4	0.04

The results indicate that the O–P–Q three-waveguide configuration will exhibit different phase accumulations for various coupling ratios *a*. We also measured the accumulated phase generated by phase controllers with optical path lengths of 5.8 mm and 6 mm, respectively, finding minimal phase differences between them. This demonstrates that the deterministic arbitrary-phase-controlling is achieved and the fabrication error resistance of the phase controller within a certain range of length. The periodic variation of phase value and optical field intensity in the waveguide Q, as shown in Figure 1(d), allows to theoretically derive the length range of phase preservation. Experimentally, we measured the positions of the first and second occurrences of maximum optical field intensity in the waveguide Q at 1.7 mm and 5.8 mm, respectively. Based on this, it can be inferred that the phase controller can achieve a phase shift within approximately a 1 mm range, which is further verified by numerical simulation in the Supplementary Information V. This phase control significantly enhances tolerance to fabrication errors in the micron/nano scale in the field of micro/nano-optics, improving the tolerance of errors by three orders of magnitude, which is demonstrated theoretically in Supplementary Information III, and the further simulation results are shown in Supplementary Information V and VI. In addition, as calculated in the Supplementary Information III, setting *a* = 0 corresponds to the absence of coupling strength in the O–Q dual-waveguide configuration. In this case, the O–P–Q three-waveguide configuration degenerates into the O–P and P–Q dual-waveguide configurations, accumulating a geometric phase of *π*. This aligns with the previous discussion.

### 2.2 Permutation circuits

**Figure 3:**
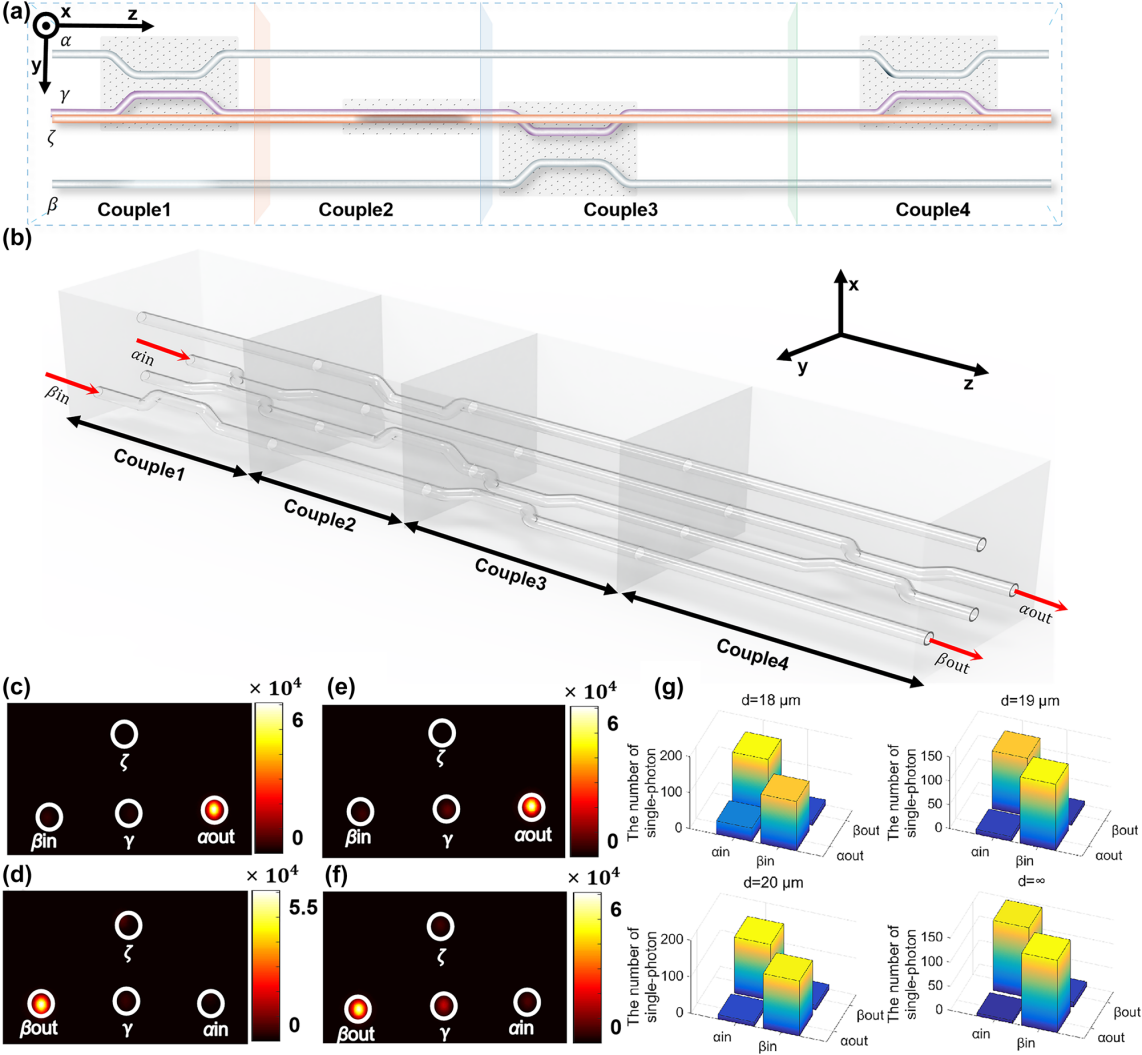
The permutation lines and experimental results. (a) The controlling of light in the permutation circuit. All four configurations Couple1, Couple2, Couple3, and Couple4 are shown; (b) the 3D structure of the permutation circuit; (c), (d) the experimental results of the permutation circuit shown in Figure 3(a), with input light from the waveguide *α* and *β*, respectively; (e), (f) the experimental results of the permutation structure with the auxiliary waveguide *ξ*; (g) the experimental results of single-photon incident the permutation circuit with input light from the waveguide *α* or *β*, which demonstrates the circuit can still realize the permutation function when the single photon is injected with different structure parameter *d*.

Based on the on-chip arbitrary-phase controller, we constructed an on-chip optical permutation circuit, shown in Figure 3(a). The main component of the circuit consists of four waveguides, labeled *α*, *β*, *γ*, and *ζ*. Waveguides *α*, *γ*, and *β* lie in the same plane during the initially and final evolved states, while waveguide *ζ* is in a different plane. This structure appears as a “T” shape in cross section, and the three-dimensional (3D) structure of the permutation circuit is depicted in Figure 3(b). The controlling of light in the permutation circuit shown in Figure 3(a) involves four different coupled controlling processes, denoted as configurations Couple1, Couple2, Couple3, and Couple4. Couple1 consists of waveguides *α* and *γ* forming O–Q dual-waveguide configuration; Couple3 involves waveguides *γ* and *β* forming O–Q dual-waveguide configuration; Couple4 comprises waveguides *α* and *γ* forming O–Q dual-waveguide configuration. Therefore, the light goes through Couple1, Couple3, and Couple4 will accumulate a geometric phase of *π*/2 each time. In contrast, configuration Couple2 involves longer O–Q dual-waveguide configuration consisting of waveguides *γ* and *ζ*. Light is first incident from waveguide *γ* and evolves to waveguide *ζ*, then evolves back from waveguide *ζ* to waveguide *γ*. This process is equivalent to undergoing two coupling processes of the O–Q dual-waveguide configuration, similar to the controlling discussed earlier in the cascaded O–P and P–Q dual-waveguide configurations, resulting in a geometric phase of *π*.

In this permutation circuit, the exchange of two input states can be achieved. The light incident from waveguide *α* will undergo a three-coupling process including Couple1, Couple2, and Couple3 and eventually be output from waveguide *β*. This process is abbreviated as *α*-1-2-3-*β*. On the other hand, light incident from waveguide *β* will undergo a two-coupling process including Couple3 and Couple4 and be output from waveguide *α*. This process is abbreviated as *β*-3-4-*α*. Therefore, the two processes will result in a phase difference of *π*/2. This fundamental circuit, composed of the O–Q dual-waveguide configuration and cascaded O–P and P–Q dual-waveguide configurations, can accumulate an integer number of *π*/2 geometric phases. In the experiments, we fabricated the samples shown in Figure 3(b) using femtosecond laser direct writing technology. The experimental results are depicted in Figure 3(c) and (d), where it can be observed that the light incident from waveguide *α* can almost exit from waveguide *β*, and the light incident from waveguide *β* can almost exit from waveguide *α*. This indicates that the permutation process can be realized using this network. In addition, another experiment is also performed based the permutation circuit. An extra auxiliary waveguide *ξ* is introduced in the Couple1, then the waveguide *α*, *ξ*, *γ* in the Couple1 process consists an O–P–Q three-waveguide configuration, which will support the deterministic arbitrary phase. Figure 3(e) and (f) shows the experimental results of another permutation structure with the auxiliary waveguide *ξ*. It can be found the auxiliary waveguide *ξ* almost no effect on the intensity in the permutation circuit. The experimental results of single-photon incident the permutation circuit are shown in Figure 3(g). The numbers of single photons exiting as the coupling coefficient between the waveguide *ξ* and *α* or *γ* increases are shown in Figure 3(g), and the numbers of single photons exiting in the permutation structure without the auxiliary waveguide *ξ* are shown in the Figure 3(g). The above single photon experiments show that the number of exiting photons does not change with the addition of waveguide *ξ*, and the process of permutation is always achievable based on the circuit.

### 2.3 The implementation of arbitrary-phase-controller and quantum-gate chip based on the permutation circuit

**Figure 4:**
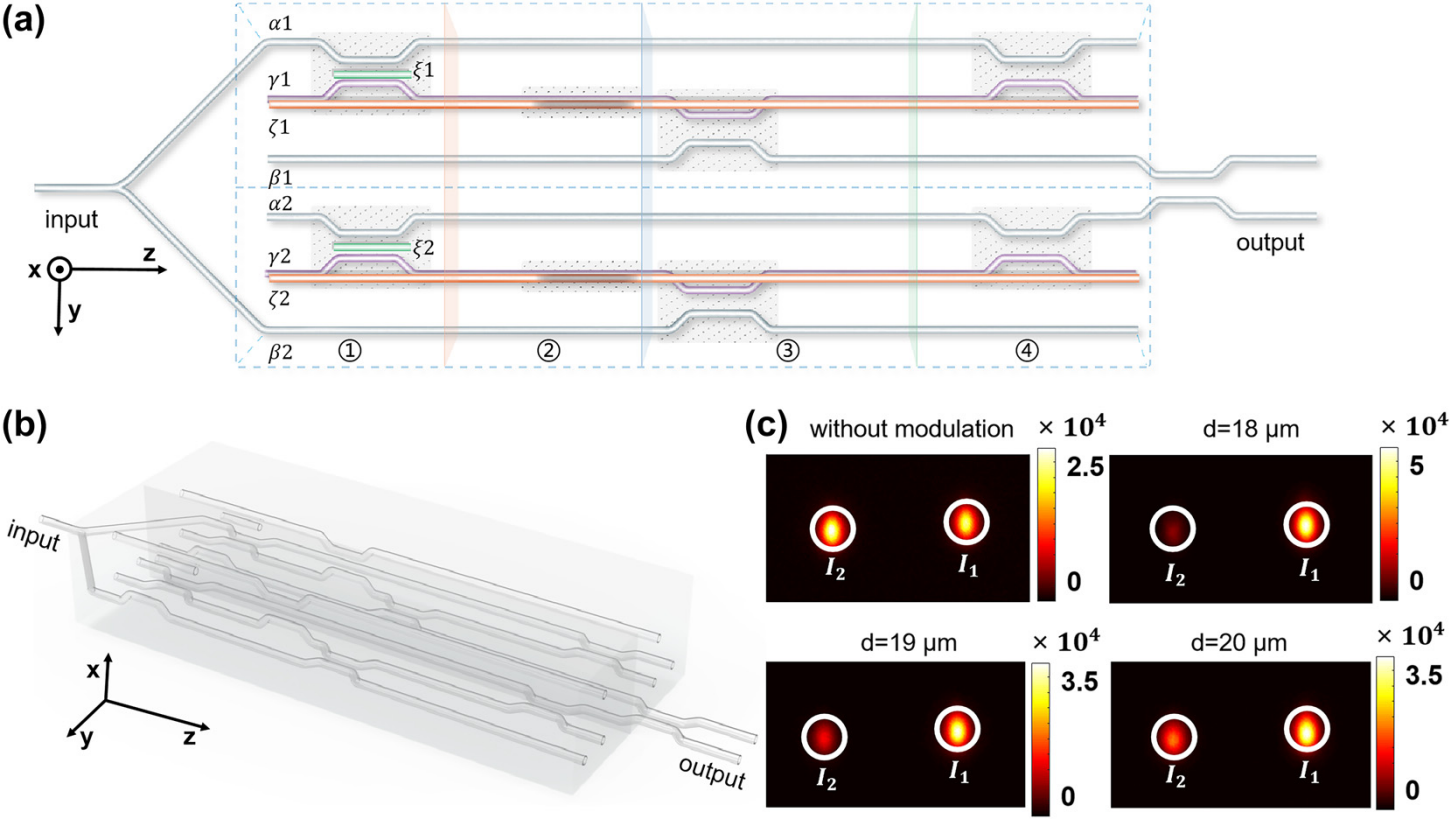
The arbitrary phase controller and quantum gate chip based on permutation circuits. (a) The permutation circuit. The number ①②③④ represents the four configurations Couple1, Couple2, Couple3, and Couple4; (b) the 3D structure of the permutation circuit; (c) the experimental results with input light from the waveguide *α*1 and *β*2 at the same time. Different phase controlling results can be obtained with different structural parameters *d* when light outputs from the waveguide *α*2 and *β*1 at the same time.

The above permutation circuit achieves the accumulation of a fixed value of *π* phase. However, by introducing the arbitrarily phase controller designed in the Section 2 into the fundamental circuit, it becomes possible to realize the permutation circuit chip with accumulation of arbitrary phases. In the coupling process of Couple1 of the fundamental circuit, we added an auxiliary waveguide *ξ*. In this case, the Couple1 process becomes an O–P–Q three-waveguide configuration, where waveguide *α* corresponds to the input waveguide O, waveguide *γ* corresponds to the output waveguide Q, and waveguide *ξ* corresponds to the control waveguide P. The permutation circuit structure is shown in Figure 4(a). In this case, the light incident from waveguide *α*1 will undergo a three-coupling process, Couple1, Couple2, and Couple3, and be output from waveguide *β*1. In the processes of Couple2 and Couple3, the light will accumulate fixed geometric phases of *π* and *π*/2 successively. The phase generated by the Couple1 process can be controlled to any arbitrary phase *θ* by adjusting the coupling ratio *a* in the *α* – *ξ* – *γ* three-waveguide system. On the other hand, the light incident from waveguide *β*2 will be output from waveguide *α*2 with a fixed *π* phase. Therefore, the entire permutation circuit can achieve phase controlling given by $\phi =\left(\pi +\frac{\pi }{2}+\theta \right)-\pi =\frac{\pi }{2}+\theta$ . Clearly, *ϕ* can be further manipulated by controlling the coupling ratio of the three-waveguide configuration.

The above permutation network was experimentally implemented using laser direct writing technology for on-chip waveguides. The schematic of the permutation circuit and its 3D structure are shown in Figure 4(b). We constructed two identical permutation circuits. In the upper circuit, the light incident from waveguide *α*1 undergoes the three-coupling process including Couple1, Couple2, and Couple3 and is output from waveguide *β*1. In the lower circuit, the light incident from waveguide *β*2 undergoes the processes of Couple3 and Couple4 and is output from waveguide *α*2. The two output beams from *β*1 and *α*2 are then injected into a 1:1 directional coupler to obtain two output beams, *I*_1_ and *I*_2_. By measuring the intensity ratio of *I*_1_ and *I*_2_, the phase difference between the two processes can be obtained. The experimental results for the output optical field intensity ratio are presented in the Table 3, and the experimental intensity ratio is shown in Figure 4(c). This permutation circuit can achieve arbitrary phase controlling during the permutation process, and the experimental results are in good agreement with theoretical calculation.

**Table 3:** The output optical field intensity distribution and the phase controlling.

*d*/μm	Theory *ϕ* (^*^ *π*)	Theory *I*_1_	Theory *I*_2_	Experimental *I*_1_	Experimental *I*_2_	Experimental *ϕ* (^*^ *π*)
18	0.25	0.85	0.15	0.82	0.18	0.25
19	0.18	0.70	0.30	0.71	0.29	0.18
20	0.04	0.56	0.44	0.55	0.45	0.05

Due to the manipulation of the input light in the permutation process, specifically for waveguides *α*1 and *β*1 (or *α*2 and *β*2), it is equivalent to manipulating the initial states of the system. This process can be described by the quantum gate $G=\left(\begin{array}{cc} 0 & 1\\ {\text{e}}^{\text{i}\phi } & 0 \end{array}\right)$ , representing that the permutation circuit can generate an arbitrary phase difference *ϕ* between the two initial states and perform a permutation operation. If the light is incident into waveguides *α*1 and *β*1 (or *α*2 and *β*2) as two quantum states $\left|0\right\rangle$ and $\left|1\right\rangle$ , the overall input quantum state of the system can be written as $\alpha \left|0\right\rangle +\beta \left|1\right\rangle$ . Through this permutation circuit with arbitrary-phase-controlling, the phase-protected manipulation of the quantum state $\alpha \left|0\right\rangle +\beta {\text{e}}^{\text{i}\theta }\left|1\right\rangle$ can be achieved. This paves the way for further development of silicon-based topological quantum computing systems in the future.

### 2.4 The on-chip silicon-based phase controller based on the three-waveguide configuration

**Figure 5:**
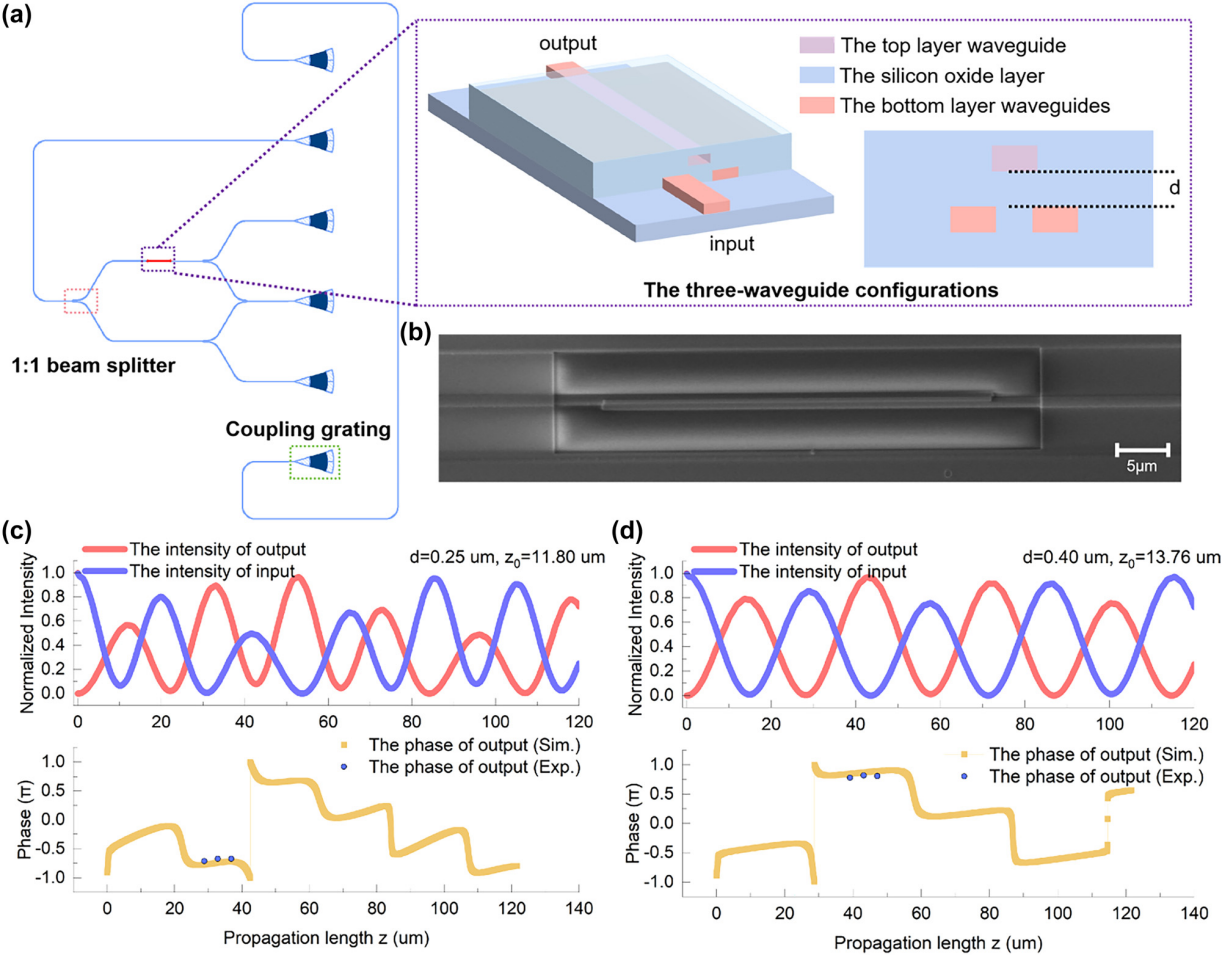
The on-chip silicon-based three-waveguide configuration phase controller. (a) The on-chip silicon phase controller based on the three-waveguide configuration. The circuit of the waveguides is shown by the blue line in the figure, and the three-waveguide configuration is shown by the red short line. The interlayer coupling coefficient between the top and bottom waveguide layer is determined by the thickness *d* of the silicon oxide layer. All three waveguides are buried in a silicon oxide layer. (b) The optical microscope image of the three-waveguide structure. (c) The scanning electron microscope image of the three-waveguide structure. (d), (e) The simulation and experimental result of the on-chip silicon phase controller based on the three-waveguide configuration when *d* = 0.25 µm and *d* = 0.4 µm. The parameter *d* corresponds to phases controlling in the three-waveguide configuration.

The three-waveguide configuration can not only be fabricated and applied to the laser direct writing platform as a phase controller but also can be further extended to the silicon-based photonic chip as a deterministic phase controller. We designed and manufactured an on-chip silicon phase controller based on the three-waveguide configuration. The complete experimental circuit of the phase controller is as shown in Figure 5(a), and the phase can be calculated by measuring the light intensity of the output [44], [45]. The phase controller consists of two silicon waveguides on a Silicon-on-Insulator (SOI) substrate and a silicon oxide layer between the waveguides. The interlayer coupling coefficient between the upper waveguide and the lower waveguide is mainly determined by the thickness of the silicon oxide layer, i.e., the thicker the silicon oxide layer, the weaker the coupling coefficient between these two layers waveguides. Figure 5(b) and (c) is the optical microscope image and the scanning electron microscope (SEM) image of the three-waveguide configuration. The simulation results of the phase controlling FDTD are shown in Figure 5(d) and (e). When the interval between the top and bottom waveguide layers is different, the simulation results show that different phase controlling can be achieved in the second and third fully transmittance regions, covering the phase range of 0–2*π*. We further verified the above results through experiments. Similar to the laser direct writing platform, the phase controlling capability of the three-waveguide configuration is determined by measuring the phase difference between the three-waveguide configuration and a single waveguide in a two-arm interferometer. As shown as Figure 5(a), before the incident phase controlling region, the injected light is evenly divided into two beams and injected into a three-waveguide phase controller and a single waveguide, respectively, and then phase controlling is performed in the three-waveguide controller. Both the light controlled through the three waveguides controller and the light that is not phase-controlled through a single waveguide are output and evenly divided into four arms, two of which are merged again so that light from the upper and lower branches can interfere. The phase can be obtained by measuring the light intensity of the three beams. Detailed results can be found in Table 4.

**Table 4:** The phase controlling for on-chip silicon phase controller based on the three-waveguide configuration.

*d*/μm	Theory *ϕ* (^*^ *π*)	The coupling length *L*/μm	Experimental *ϕ* (^*^ *π*)		
			Δ*L* = 0 μm	Δ*L* = −4 μm	Δ*L* = 4 μm
0.2	−0.7	26.36	−0.68	−0.65	−0.85
0.25	−0.75	32.80	−0.67	−0.71	−0.67
0.4	0.80	43.08	0.82	0.78	0.81

The results indicate that the O–P–Q three-waveguide configuration will exhibit different phase accumulations for various coupling ratios *a*. The coupling length *L* is the center in the second fully transmittance regions, and Δ*L* is the distance from the center *L*. We also measured the accumulated phase generated by phase controllers with optical path lengths of *L* and *L* ± Δ*L*, respectively, finding minimal phase differences between them. This demonstrates that the deterministic arbitrary-phase-controlling within a certain range of length can be achieved by the phase controller. The phase controlling capability of the three-waveguide configuration is completely determined by geometric parameters and has strong stability. The stability of the phase controller is discussed in detail in the Supplementary Information VI, and the potential to resist fabrication errors is further discussed in the Supplementary Information VI. Compared with the single-layer architecture of the previous photonic integration platform, we realize stable optical transmission and phase controlling between layers based on the two-layer photonic integration platform and expand the on-chip photonic integration to quasi-two-dimensional or even three-dimensional space. The phase controller opens a new sight to design and machine on-chip novel optical devices with higher integration and more complex functions.

## 3 Discussion

We propose and experimentally demonstrate an on-chip phase controller, which can achieve deterministic arbitrary-phase-controlling and resist against fabrication errors in the process of permutation. The phase controlling within a specific range is solely dependent on the coupling structure and remains unaffected by the fabrication errors based on the three-waveguide configurations. The three-waveguide configurations can be installed in a permutation circuit as a coupled phase controller, which can realize the phase protection manipulation of the quantum state. Through the permutation programming process, we construct a quantum gate. This research paves the way for further development of topological quantum computing systems. We also verified that the on-chip silicon-based phase controller with the three-waveguide configuration has the advantages of small size and low energy consumption, which open a new sight to design and machine on-chip novel optical devices with higher integration and more complex functions. Furthermore, our work can be combined with metasurfaces. If the upper waveguide is precisely designed to radiate light outward while still supporting guided modes, or if a metasurface structure is added above the upper waveguide, comprehensive control of the optical field can be achieved. This is accomplished through the coupling between the waveguide and the metasurface, as well as the metasurface’s ability to further manipulate the radiated light field [46], [47], [48]. These approaches enable combined control over both the guided-mode evolution governed by the lower waveguide-waveguide coupling structure and the radiation field governed by the upper metasurface-waveguide coupling structure, which brings new possibilities for controlling of the light field in the future.

## 4 Methods

The phase controlling based on the laser direct writing platform:

The three-waveguide coupling systems consisting of the single-mode waveguides are fabricated in borosilicate glass (Eagle XG, Corning) by focusing pulses generated by a regeneratively amplified Yb: KGW femtosecond laser system (Pharos20, Light Conversion) at a wavelength of 1,030 nm with a duration of 240 fs, and 1-MHz repetition rate. The microscope objective (20×, NA = 0.45) focus the pulse with a power of 330 nJ at a depth of 170 μm below the glass surface to write the two waveguides on the downer layer, while the depth of the waveguide on the upper layer is determined by the parameter d in the main text. We move samples at a speed of 30 mm/s by using a computer-controlled high-precision three-axis air-bearing stage (FG1000-150-5-25-LN, Aerotech). The propagation loss of the straight waveguide is 0.95 dB/cm.

The phase controlling based on the on-chip silicon photonic chip platform:

The three-waveguide coupling systems consisting of the single-mode waveguides are fabricated on the SOI (Silicon-On-Insulator) substrates. The 2 cm × 2 cm SOI substrates were used to fabricate different samples with different silicon oxide thickness. The bottom two waveguides are fabricated by electron beam lithography and ICP (Inductively Coupled Plasma) etching. Then the silicon oxide with different thicknesses and a 220 nm amorphous silicon layer are deposited on these two waveguides. The top layer waveguide is also fabricated by electron beam lithography and ICP etching.

## Supplementary Material

Supplementary Material Details
