# Supplementary material for: On-chip deterministic arbitrary-phase-controlling
Source: Nanophotonics. 2025 Jul 2;14(15):2633–46. doi: 10.1515/nanoph-2025-0132 (PMC12322723; doi:10.1515/nanoph-2025-0132)
Supplement: Supplementary file 1 — Supplementary Material Details [file j_nanoph-2025-0132_suppl_001.pdf]

Supporting Information

On-chip deterministic arbitrary-phase-controlling

Rui Ma,[1,#] Chu Li,[1,#] Qiuchen Yan,[1,#,] Xinyi Wang,[1,#] Ruiqi Wang,[1] Yufei Wang,[1] Yumeng Chen,[1] Yan Li,[1,2,3,4,*] Cuicui Lu,[5,*] Jianwei Wang,[1,2,3,4,*] Xiaoyong Hu,[1,2,3,4,*] C.T. Chan,[6,*] and Qihuang Gong[1,2,3,4]*

[1]State Key Laboratory for Mesoscopic Physics & Department of Physics, Collaborative Innovation Center of Quantum Matter & Frontiers Science Center for Nano-optoelectronics, Peking University, Beijing 100871, P. R. China

[2]Peking University Yangtze Delta Institute of Optoelectronics, Nantong, Jiangsu 226010, P. R. China

[3]Collaborative Innovation Center of Extreme Optics, Shanxi University, Taiyuan, Shanxi 030006, P. R. China

[4]Hefei National Laboratory, Hefei 230088, P. R. China

[5]Laboratory of Advanced Optoelectronic Quantum Architecture and Measurements of Ministry of Education, Beijing Key Laboratory of Nanophotonics and Ultrafine Optoelectronic Systems, Center for Interdisciplinary Science of Optical Quantum and NEMS Integration, School of Physics, Beijing Institute of Technology, Beijing 100081, P. R. China

[6]Department of Physics and Institute for Advanced Study, The Hong Kong University of Science and Technology, Clear Water Bay, Kowloon, Hong Kong, China

[#] The authors contributed equally to this work.

*E-mail: qiuchenyan@pku.edu.cn, li@pku.edu.cn, cuicuilu@bit.edu.cn, jianwei.wang@pku.edu.cn, xiaoyonghu@pku.edu.cn, phchan@ust.hk

Contents

I. The advantage of the arbitrary-phase-controller

II. The phase analyses and comparison between the dual-waveguide configurations and the three-waveguide configuration

III. The theory of on-chip arbitrary-phase-controller resisting the fabrication error

IV. The relationship of waveguide coupling strength with waveguide spacing in the experiment based on the laser direct writing platform

V. The extra result of the experiment based on the laser direct writing platform.

VI. The extra result of the experiment and verification of the stability of the on-chip silicon-based three-waveguide configurations.

VII. The schematic of the optical measurement setup.

I. The advantage of the arbitrary phase controller.

In this work, we have proposed an effective strategy for on-chip deterministic arbitrary phase controlling. With our method, the phase controlling ranges from 0 to π can be achieved theoretically, and the phase controlling range from 0 to π/4 is proved experimentally by femtosecond laser direct writing method. Different from those traditional methods based on MZI and MRR, our method has excellent phase fabrication error resistance. The "capacity of resisting fabrication error" refers to the robustness to the machining length error when preparing the structure. Specifically, the length change of the three-waveguide structure does not affect the phase controlling in a certain range because the arbitrary-phase-controller is achieved. Our experiment demonstrates the fabrication error resistance of the phase controller within a certain range of length in the section 2 and section 5. We compare our work with existing reports of the other phase controlling in the main text of Table 1**.**

We also summarize and detailed the novelty and innovative points of our work in following Figure S1.

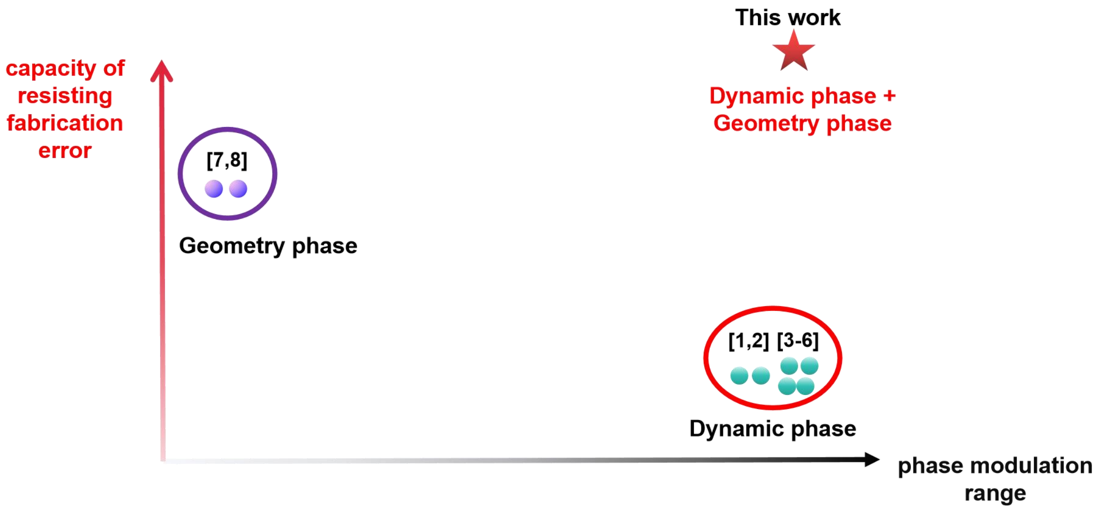

Figure S1. The advantages of the phase controller

Reference:

[1] F. Ceccarelli, S. Atzeni, C. Pentangelo, F. Pellegatta, A. Crespi, R. Osellame, *Laser Photonics Rev.* **2020**, *14*, 2000024.

[2] F. Flamini, L. Magrini, A. S. Rab, N. Spagnolo, V. D'Ambrosio, P. Mataloni, F. Sciarrino, T. Zandrini, A. Crespi, R. Ramponi, R. Osellame, *Light Sci Appl* **2015**, *4*, e354.

[3] S. Pai, Z. Sun, T. W. Hughes, T. Park, B. Bartlett, I. A. D. Williamson , M. Minkov, M. Milanizadeh, N. Abebe, F. Morichetti, A. Melloni, S. Fan, O. Solgaard, D. A. B. Miller, *Science* **2023**, *380*, 398-404.

[4] J. Bütow, J. S. Eismann, V. Sharma, D. Brandmüller, P. Banzer, *Nat. Photon.* **2024**, *18*, 243-249.

[5] D. D. Bühler, M. Weiß, A. Crespo-Poveda, E. D. S. Nysten, J. J. Finley, K. Müller, P. V. Santos, M. M.

de Lima Jr., H. J. Krenner, *Nat Commun* **2022**, *13*, 6998.
[6] N. Sun, D. Sun, D. Wu, Y. Guo, Y. Fan, F. Zou, M. Pu, X. Luo, *Laser Photonics Rev* **2024**, *18*, 2300937.
[7] X. Zhang, F. Yu, Z. Chen, Z. Tian, Q. Chen, H. Sun, G. Ma, *Nat. Photon*. **2022,** *16*, 390-395.
[8] W. Song, S. Wu, Y. Chen, C. Chen, S. Gao, C. Huang, K. Qiu, S. Zhu, T. Li, *Phys. Rev. Applied* **2022**, *17*, 014039.

The on-chip deterministic arbitrary phase controlling based on the three-waveguide coupled configuration has the enhanced resistance against fabrication errors, which is possible to achieve large-scale integration and the quantum gate operations in optical permutation-group circuits. We show the substantial advantage with respect to solutions in Figure S2, and show the splendid resistance against fabrication errors of our research from origin in Table S1.

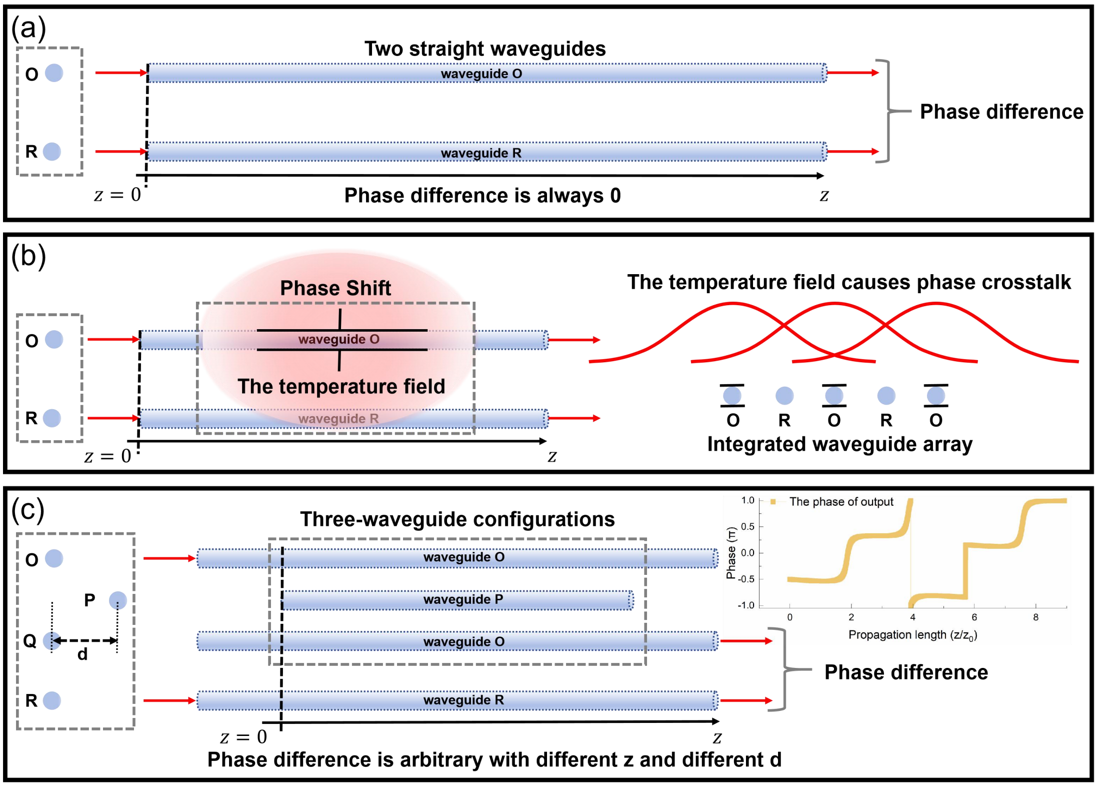

Figure S2. The comparison between different phase controllers. (a) The dual independent waveguides, which is hard to produce a phase difference at same propagation length. (b) The conventional thermo-optical phase controller. The phase crosstalk exists in this controller caused by the temperature field. The red area shows the distribution of the temperature field of the thermo-optical phase controller. (c) The three-waveguide configurations. The phase difference is arbitrary with different z and different d in the phase controller.

We further show the substantial advantage with respect to solutions in Figure S2. For two identical waveguides, it is hard to produce a phase difference at same propagation length because the two waveguides will accumulate the same dynamic phase, as shown as Figure

S2(a). Therefore, although the dual independent waveguide structure shown in Figure S2(a) can ensure the phase stability under the same propagation length, it will lose the ability to control the phase difference. The three waveguide configuration is shown as Figure S2(c). The different phases can be realized at the same transmission length *z* by adjusting the structural parameter *d* in the three-waveguide configuration. At the same time, at the transmission positions corresponding to different phase platform, the phase controller can also realize different phase control with the same structural parameter *d*. Moreover, it can be verified that the three-waveguide configuration are more resistant to error than single waveguide earlier. These are advantages that the dual independent waveguides don't have.

We also compare our phase controller with the conventional phase controller. For example, the thermo-optical phase controller is currently used on both femtosecond direct writing platforms and silicon-based platforms, as shown in Figure S2(b). The thermal optical phase controller changes the optical field phase by heating the waveguide to change its refractive index. Heating waveguide will produce a wide temperature field near the corresponding waveguide. If the gap between the waveguide is large to avoid phase crosstalk generated by the temperature field, it will lead to a decrease in chip integration. However, if the distance between waveguides is reduced, the phase crosstalk caused by temperature field is inevitable, which makes it difficult to calibrate each phase controller during the experiment. Although it has been shown that the effect of thermal crosstalk can be reduced by preparing thermal isolators[17], it makes the preparation of photonic chips more complicated. The phase controller based on the three-waveguide configuration solves the problem of thermal crosstalk from the root because the phase controller does not contain a heating electrode. This is our advantage over traditional phase controller.

Table S1. Comparison of fabrication tolerance

Phase controller	Fabrication tolerance	Fabrication technique
Straight waveguide	0 nm	Electron beam lithography
MZI	0 nm	Electron beam lithography
MRR	0.004-fold the radius (about 20nm for 5-μm radius)	Electron beam lithography
Our work	About 1 mm	Laser direct writing
	About 10 μm	Electron beam lithography

For the straight waveguide phase controller, the phase controlling is on the basis of the

dynamic phase, depending on the waveguide length. Therefore, the fabrication tolerance of the straight waveguide is 0 nm. For the MZI phase controller without the electro- or thermal- optical tuning, the phase controlling is on the basis of the dynamic phase, depending on the waveguide length. Therefore, the fabrication tolerance is 0 nm. For the MRR phase controller without the electro- or thermal- optical tuning, the phase controlling is on the basis of the resonance response, depending on the ring radius. The fabrication tolerance is 0.004-fold the radius (about 20nm for 5-μm radius). With the electro- or thermal- optical tuning, MZI and MRR phase controller has a large feature size. The serious crosstalk is unavoidable in practical large-scale integration application.

Last, the phase controller based on the three-waveguide configuration also has the ability to control light intensity at the same time. As shown as Figure. 1(c)(d) in the main text, for the same phase platform, the normalized light intensity gradually increases from 0 to 1 and then returns to 0 during the platform period. At different positions z in the same plateau platform, the phase controller embodies the ability of light intensity control. For different phase platform, the phase is different, but the intensity in the same platform period follows the same change law. This shows that in the phase controller, the intensity and phase are two independent dimensions. By selecting different transmission lengths z and structure parameter d, the light intensity and phase can be controlled simultaneously. This further shows that the three-waveguide configuration can not only be used as a phase controller, but also has the ability of control light intensity.

II. The phase analyses and comparison between the dual-waveguide configurations and the three-waveguide configuration.

In our work, we present an effective strategy utilizing a three-waveguide coupling configuration to realize on-chip deterministic arbitrary phase controlling. Here, we make a comparison between the dual-waveguide configurations and the three-waveguide configuration as shown as the Figure S3. The Figure S3(a) shows the three-waveguide configuration realizing on-chip deterministic arbitrary phase controlling. However, the dual-waveguide configuration only realizes on-chip deterministic π/2 phase controlling because the phase shift of π/2 is only introduced in the evolution process of this dual-waveguide configuration.

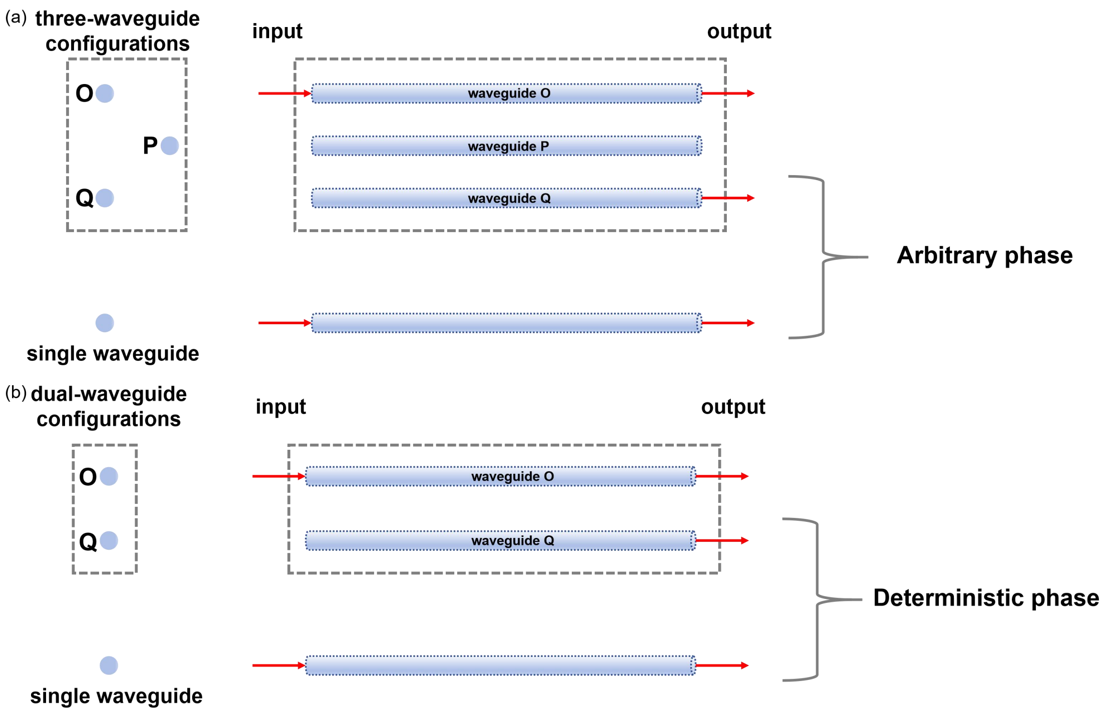

Figure S3. (a) the three-waveguide configuration which realizes on-chip deterministic arbitrary phase controlling; (b) the dual-waveguide configuration which only realizes on-chip deterministic $\pi/2$ phase controlling.

The evolution process of the three-waveguide configuration and dual-waveguide configuration are analyzed in the research. The Figure S4 shows the optical field intensity and the phase distributions in the two configurations. The difference of phase between the waveguide O and waveguide Q are obtained as shown in Figure S4(c) and Figure S4(e).

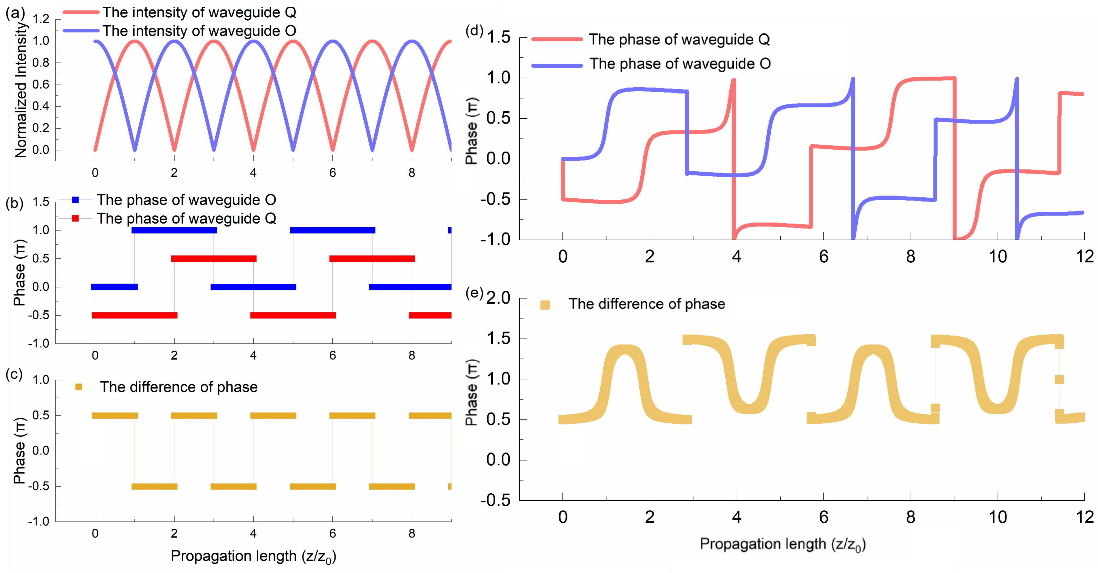

Figure S4. (a)(b) the optical field intensity and the phase distributions in waveguide O, waveguide Q in the

dual-waveguide configuration; (c) the difference of phase between the waveguide O and waveguide Q in the dual-waveguide configuration; (d) the optical field phase in waveguide O, waveguide Q in the three-waveguide configuration; (e) the difference of phase between the waveguide O and waveguide Q in the three-waveguide configuration.

III. The theory of the on-chip arbitrary-phase-controller resisting the fabrication error.

In the text, we introduce the on-chip deterministic arbitrary phase controller resisting the fabrication error based on a three-waveguide system. The theory of the two identical dual-waveguide configurations and the three-waveguide configuration are described in detail in the main text. Here, the theory of the deterministic arbitrary phase controller will be given in detail.

The Hamiltonian for the three-waveguide system is given by $H = \begin{pmatrix} 0 & \kappa_1 & \kappa_2 \\ \kappa_1 & 0 & \kappa_1 \\ \kappa_2 & \kappa_1 & 0 \end{pmatrix}$ in the main text, simply obtaining the eigenvalues and eigenvectors of the system, seen from Equation (3) (4) (5) in the main text.

$$\begin{cases} \psi_1 = (-1,0,1)^T & \lambda_1 =- \kappa_2 \quad (3) \\ \psi_2 = \left(1, -\dfrac{4\kappa_1{}^2 - \kappa_2{}^2 - \kappa_2\sqrt{8\kappa_1{}^2 + \kappa_2{}^2}}{\kappa_1\left(-3\kappa_2 + \sqrt{8\kappa_1{}^2 + \kappa_2{}^2}\right)}, 1\right)^T & \lambda_2 = \dfrac{1}{2}\left(\kappa_2 - \sqrt{8\kappa_1{}^2 + \kappa_2{}^2}\right) \quad (4) \\ \psi_3 = \left(1, -\dfrac{-4\kappa_1{}^2 + \kappa_2{}^2 - \kappa_2\sqrt{8\kappa_1{}^2 + \kappa_2{}^2}}{\kappa_1\left(3\kappa_2 + \sqrt{8\kappa_1{}^2 + \kappa_2{}^2}\right)}, 1\right)^T & \lambda_3 = \dfrac{1}{2}\left(\kappa_2 + \sqrt{8\kappa_1{}^2 + \kappa_2{}^2}\right) \quad (5) \end{cases}$$

If light is incident into waveguide O at where *z* is equal to zero, the initial state vector can be expressed as $\varphi_{z=0} = (1,0,0)^T$. Expanding it in terms of the eigenvectors, it takes the form as $\varphi_{z=0} = A\psi_1 + B\psi_2 + C\psi_3$. It can be calculated that the parameters $A = \frac{1}{2}$, $B = \frac{1}{2+\left[\frac{4\kappa_1{}^2-\kappa_2{}^2-\kappa_2\sqrt{8\kappa_1{}^2+\kappa_2{}^2}}{\kappa_1\left(3\kappa_2-\sqrt{8\kappa_1{}^2+\kappa_2{}^2}\right)}\right]^2}$, and $C = \frac{1}{2+\left[\frac{4\kappa_1{}^2-\kappa_2{}^2+\kappa_2\sqrt{8\kappa_1{}^2+\kappa_2{}^2}}{\kappa_1\left(3\kappa_2+\sqrt{8\kappa_1{}^2+\kappa_2{}^2}\right)}\right]^2}$.

For simplicity, we introduce the parameter $a = \frac{\kappa_2}{\kappa_1}$ to represent the ratio between the two different coupling strengths. By substituting $\kappa_2 = a\kappa_1$ into the previous expression, and replacing A 、 B 、 C 、 ψ_1 、 ψ_2 、 ψ_3 , the evolution function for the state vector from waveguide O in the O-P-Q three-waveguide system can be obtained. Furthermore, the evolution process of the state vector in the three-waveguide system can be expressed as

Equation (6) shows.

$$\varphi_z = A\psi_1 e^{-i\lambda_1 z} + B\psi_2 e^{-i\lambda_2 z} + C\psi_3 e^{-i\lambda_3 z} \#(6)$$

With the mathematical substitution$\kappa_2 = a\kappa_1$, the evolution process of the state vector could be written as Equation (7):

$$\varphi_z =-\frac{1}{2}\begin{pmatrix}-1\\0\\1\end{pmatrix}e^{i\kappa_2 z}$$

$$+\begin{pmatrix}\frac{5a^2-3a\sqrt{8+a^2}+4}{10a^2+16+a^4-(10a-a^3)\sqrt{8+a^2}}\\ \frac{5a^2-3a\sqrt{8+a^2}+4}{10a^2+16+a^4-(10a-a^3)\sqrt{8+a^2}}\cdot\frac{4-a^2-a\sqrt{8+a^2}}{3a-\sqrt{8+a^2}}\\ \frac{5a^2-3a\sqrt{8+a^2}+4}{10a^2+16+a^4-(10a-a^3)\sqrt{8+a^2}}\end{pmatrix}e^{-i\frac{1}{2}\left(a-\sqrt{8+a^2}\right)\kappa_1 z}$$

$$+\begin{pmatrix}\frac{5a^2+3a\sqrt{8+a^2}+4}{10a^2+16+a^4+(10a-a^3)\sqrt{8+a^2}}\\ \frac{5a^2+3a\sqrt{8+a^2}+4}{10a^2+16+a^4+(10a-a^3)\sqrt{8+a^2}}\cdot\frac{4-a^2+a\sqrt{8+a^2}}{3a+\sqrt{8+a^2}}\\ \frac{5a^2+3a\sqrt{8+a^2}+4}{10a^2+16+a^4+(10a-a^3)\sqrt{8+a^2}}\end{pmatrix}e^{-i\frac{1}{2}\left(a+\sqrt{8+a^2}\right)\kappa_1 z}$$

The following relationship can be proved mathematically:

$$\frac{5a^2-3a\sqrt{8+a^2}+4}{10a^2+16+a^4-(10a-a^3)\sqrt{8+a^2}}+\frac{5a^2+3a\sqrt{8+a^2}+4}{10a^2+16+a^4+(10a-a^3)\sqrt{8+a^2}}=\frac{1}{2}$$

$$\frac{5a^2-3a\sqrt{8+a^2}+4}{10a^2+16+a^4-(10a-a^3)\sqrt{8+a^2}}\cdot\frac{4-a^2-a\sqrt{8+a^2}}{3a-\sqrt{8+a^2}}$$

$$+\frac{5a^2+3a\sqrt{8+a^2}+4}{10a^2+16+a^4+(10a-a^3)\sqrt{8+a^2}}\cdot\frac{4-a^2+a\sqrt{8+a^2}}{3a+\sqrt{8+a^2}}=0$$

For the above formula, it can also be transformed into the following form, which is relatively simple. The mathematical substitutions are introduced:

$$J=\frac{5a^2-3a\sqrt{8+a^2}+4}{10a^2+16+a^4-(10a-a^3)\sqrt{8+a^2}}\cdot\frac{4-a^2-a\sqrt{8+a^2}}{3a-\sqrt{8+a^2}}$$

$$K=\frac{5a^2-3a\sqrt{8+a^2}+4}{10a^2+16+a^4-(10a-a^3)\sqrt{8+a^2}}$$

And then Equation (7) can be simplified:

$$\varphi_z=e^{i\frac{1}{4}\left(a-\sqrt{8+a^2}\right)\kappa_1 z}\begin{pmatrix}cos\left(\frac{3}{4}a\kappa_1 z+\frac{1}{4}\kappa_1\sqrt{8+a^2}z\right)\\0\\-isin\left(\frac{3}{4}a\kappa_1 z+\frac{1}{4}\kappa_1\sqrt{8+a^2}z\right)\end{pmatrix}$$

$$+\begin{pmatrix}K\\J\\K\end{pmatrix}e^{-i\frac{1}{2}a\kappa_1 z}2isin\left(\frac{1}{2}\kappa_1\sqrt{8+a^2}z\right)$$

Then the evolution functions of the optical fields in waveguides O and Q from φ_z are obtained:

$$O(a,\kappa_1,z)=cos\left(\frac{3}{4}a\kappa_1 z+\frac{1}{4}\kappa_1\sqrt{8+a^2}z\right)e^{\frac{1}{4}i\kappa_1\left(a-\sqrt{8+a^2}\right)z}$$

$$+\frac{5a^2-3a\sqrt{8+a^2}+4}{10a^2+16+a^4-(10a-a^3)\sqrt{8+a^2}}e^{-\frac{1}{2}ia\kappa_1 z}\cdot 2sin\left(\frac{1}{2}\kappa_1\sqrt{8+a^2}z\right) \quad (7)$$

$$Q(a,\kappa_1,z)=-isin\left(\frac{3}{4}a\kappa_1 z+\frac{1}{4}\kappa_1\sqrt{8+a^2}z\right)e^{\frac{1}{4}i\kappa_1\left(a-\sqrt{8+a^2}\right)z}$$
$$+\frac{5a^2-3a\sqrt{8+a^2}+4}{10a^2+16+a^4-(10a-a^3)\sqrt{8+a^2}}e^{-\frac{1}{2}ia\kappa_1 z}$$
$$\cdot 2sin\left(\frac{1}{2}\kappa_1\sqrt{8+a^2}z\right) \quad (8)$$

In fact, with the Equation (7), the relationship between phase value and parameters a, κ_1, and z can also been obtained:

$$\varphi=arctan\{-\left\{\frac{1}{2}sin(a\kappa_1 z)\right.$$
$$+\frac{5a^2-3a\sqrt{8+a^2}+4}{10a^2+16+a^4-(10a-a^3)\sqrt{8+a^2}}sin\left[\frac{1}{2}\left(a-\sqrt{8+a^2}\right)\kappa_1 z\right]$$
$$\left.+\frac{5a^2+3a\sqrt{8+a^2}+4}{10a^2+16+a^4+(10a-a^3)\sqrt{8+a^2}}sin\left[\frac{1}{2}\left(a-\sqrt{8+a^2}\right)\kappa_1 z\right]\right\}$$

$$/\left\{-\frac{1}{2}cos(a\kappa_1 z)+\frac{5a^2-3a\sqrt{8+a^2}+4}{10a^2+16+a^4-(10a-a^3)\sqrt{8+a^2}}cos\left[\frac{1}{2}\left(a-\sqrt{8+a^2}\right)\kappa_1 z\right]\right.$$
$$\left.+\frac{5a^2+3a\sqrt{8+a^2}+4}{10a^2+16+a^4+(10a-a^3)\sqrt{8+a^2}}cos\left[\frac{1}{2}\left(a-\sqrt{8+a^2}\right)\kappa_1 z\right]\right\}\}$$

Further, a degradation explain should also be given. With setting $a = 0$ corresponds to the absence of coupling strength in the O-Q dual waveguide configuration, the O-P-Q three-waveguide system degenerates into the O-P and P-Q dual-waveguide configurations, accumulating a geometric phase of π. The instructions are given here. With setting $a = 0$, the Equation (7) degenerates as:

$$\varphi_z = e^{-i\frac{\sqrt{2}}{2}\kappa_1 z}\begin{pmatrix} cos\left(\frac{1}{2}\kappa_1\sqrt{2}z\right) \\ 0 \\ -isin\left(\frac{1}{2}\kappa_1\sqrt{2}z\right)\end{pmatrix} + \begin{pmatrix} K \\ J \\ K \end{pmatrix}\cdot 2isin\left(\kappa_1\sqrt{2}z\right)$$

And it can be calculated that $K = \frac{1}{4}$ and $J =-\frac{\sqrt{2}}{4}$ when setting $a = 0$. According to the condition of the light completely transfers from waveguide O to waveguide Q: $\frac{1}{2}\kappa_1\sqrt{2}z = \frac{\pi}{2}$, it is easy to get $\varphi_z = \begin{pmatrix} 0 \\ 0 \\ -1 \end{pmatrix}$. This is consistent with the conclusion mentioned above.

Further, the influence of dynamic phase on the three-waveguide phase controller can be discussed. The propagation constant of the waveguide is set to 0 in the previous discussion. If a non-zero propagation constant is introduced into the phase controller, the dynamic phase will affect the phase. It is well known that if the propagation constant in a straight waveguide is a constant, then the dynamic phase will accumulate uniformly and continuously as the light propagates through the waveguide. Therefore, if a non-zero propagation constant is introduced into the three-waveguide configuration, the original plateau will tilt under the influence of the dynamic phase. For example, a non-zero propagation constant is introduced into waveguide Q to analyze the effect of the dynamic phase when the propagation constant of waveguide O and P are 0. We calculated the distribution of output optical field intensity and phase caused by the waveguide Q propagation constant $\beta = 1$ when $\kappa_1 = 1$ and $a = 3$ as shown as Figure S5(a)(b). It is can be found that the original phase platform has been tilted after considering the larger propagation constant. Figure S5(c)(d) shows the phase distribution of a three-waveguide with $\beta = 0$ and a single waveguide with $\beta = 1$, respectively. The phase change shown in Figure S5(b) is the superposition of the two phase changes shown in Figure S5(c)(d), which shows that the waveguide propagation constant affects the slope of the phase platform.

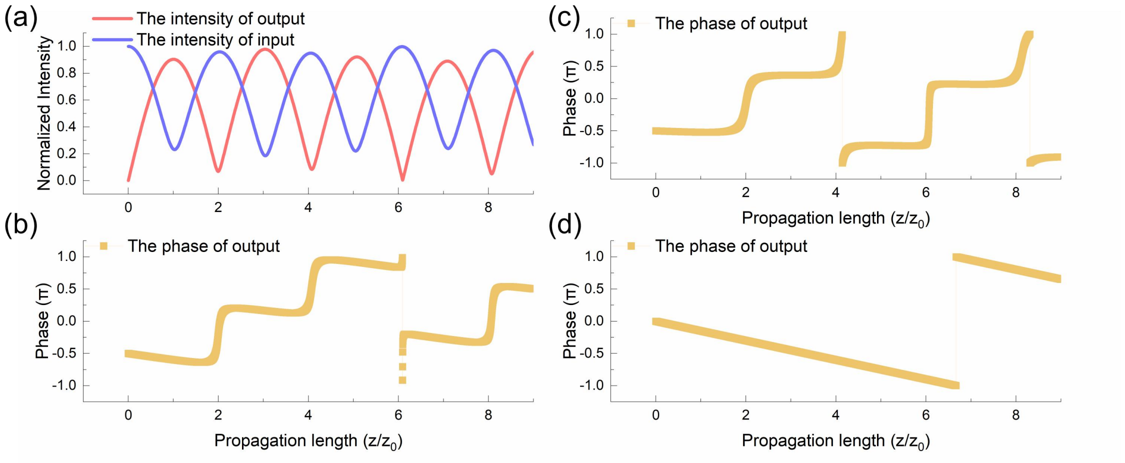

Figure S5. The influence of a non-zero propagation constant in the three-waveguide configuration. (a)(b) The distribution of output optical field intensity and phase caused by the waveguide Q propagation constant $\beta = 1$ when $\kappa_1 = 1$ and $a = 3$. (c) The phase distribution of a three-waveguide with $\beta = 0$. (d) The phase distribution of a single waveguide with $\beta = 1$.

It can be further proved that the influence of the propagation constant on the three-waveguide configuration is less than that on the single waveguide through theoretical calculation and simulation, as shown in Figure S5(b)(d), because the slope of the single-waveguide phase distribution is significantly greater than that of the three-waveguide configuration. The slope of the single-waveguide phase distribution is -1, and the slope of the three-waveguide configuration phase distribution is about -0.5. Because the three waveguides are coupled together as a whole, the change in the propagation constant β of a single waveguide will be averaged out by the multi-waveguide system, so that the final effect on the three-waveguide system is weaker than that on the single waveguide. This further demonstrates that multi-waveguide coupled systems are more resistant to error than single waveguide systems.

The effect of various fabrication errors on the three-waveguide configuration is also further discussed in the supplementary information V and VI. In the main text, we discuss the influence of the waveguide length fabrication error on the three-waveguide configuration due to the appearance of the plateau. In the following, we will further discuss the influence of the fabrication error of the waveguide radius on the laser direct writing platform and the relative position of the double-layer waveguide on the silicon-based platform, and further explain the tolerance of our device to the preparation error.

IV. The relationship of waveguide coupling strength with waveguide spacing in the experiment based on the laser direct writing platform.

In our work, the phase-controlling range from 0 to π/4 is demonstrated experimentally by using femtosecond laser direct writing method. The coupling coefficient between waveguides is calibrated by experiment in the experiment. The fitted coupling coefficient κ as a function of the gap distance d between adjacent waveguides is shown in the Figure S6.

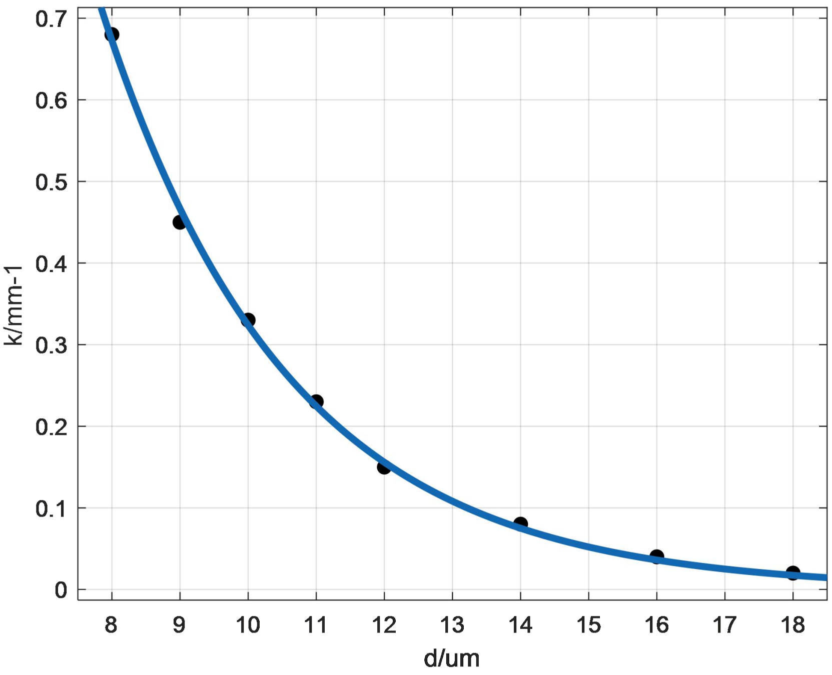

Figure S6. Fitted coupling coefficient κ as a function of the gap distance d between adjacent waveguides.

V. The extra result of the simulation and experiment based on the laser direct writing platform.

In our work, we verified the phase controlling capability of the three-waveguide configurations the resistance to fabrication errors based on the laser direct writing platform. Here, the simulation results about the three-waveguide configurations are shown as following to show the stronger phase controlling capability and the resistance to fabrication errors based on the laser direct writing platform (by using Beamlab software). We present the distribution of output optical field intensity and phase in the three-waveguide configurations as Figure S7. It can be found that the intensity of the optical field in the three-waveguide structure changes periodically, but the phase maintains the plateau characteristics. The Figure S7(a) shows the distribution of the optical field intensity in the three-waveguide configurations at y=0 (The controlling waveguide P is not on the cross-section y=0, so the controlling waveguide P is not visible in the Figure S7(a)), and the Figure S7(b)-(e) shows the phase distribution in the three-waveguide configurations with different structural parameter d. The simulation results show

that the three-waveguide configurations can realize stronger phase controlling from 0.36π to 0.6π and has a phase plateau of about 1.5 mm, which is consistent with the results predicted by our theory.

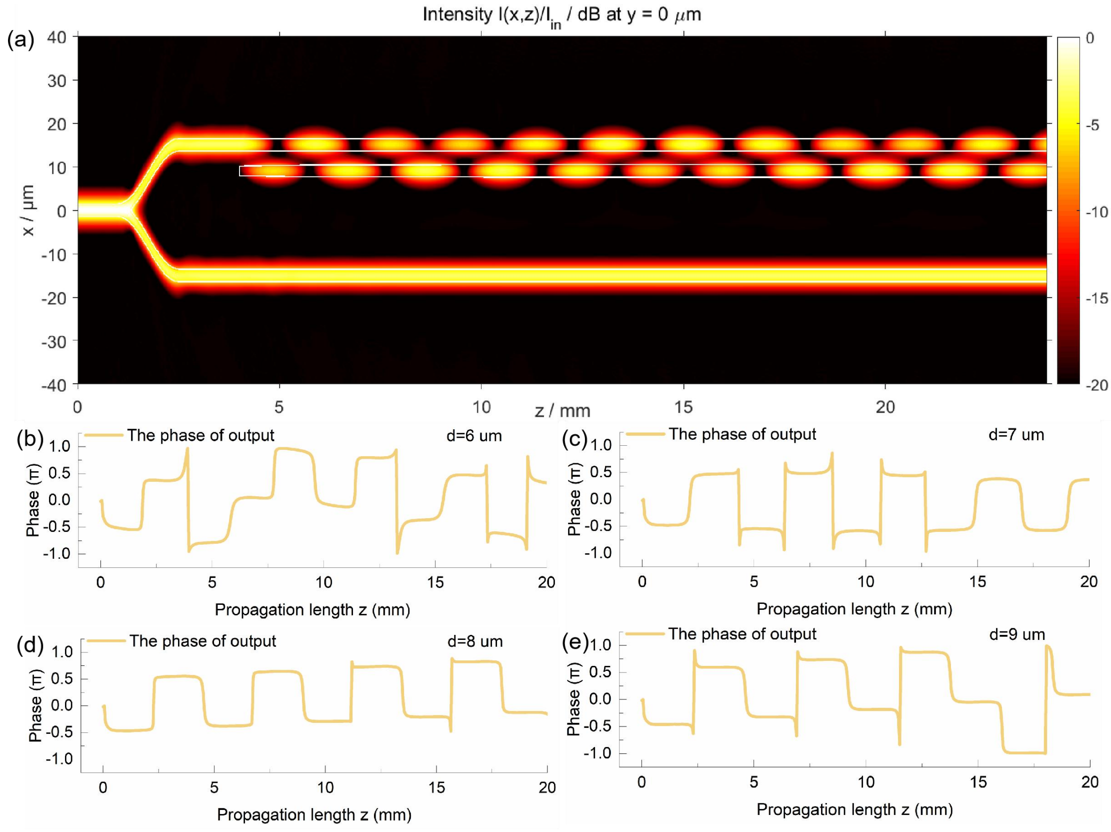

Figure S7. The simulation results about the three-waveguide configurations based on the laser direct writing platform. (a) The distribution of the optical field intensity in the three-waveguide configurations at y=0 when d=6 μm. (b)(c)(d)(e) The simulation phase result of the three-waveguide configurations when d=6 μm, d=7 μm, d=8 μm, d=9 μm, respectively. The simulation phase results show the stronger phase controlling from 0.36π to 0.6π and has a phase plateau of about 1.5 mm.

The resistance to fabrication errors of the three-waveguides configurations is also further verified by simulation. The dynamic phase error is caused by the difference of waveguide propagation constant between the two arms of the interferometer, and the difference of waveguide propagation constant is a small amount relative to the propagation constant of each waveguide itself. Therefore, the dynamic phase difference induced by the propagation constant difference is very small in our three-waveguide coupling configuration. The supplementary calculation and simulation are carried out to illustrate the impact of inevitable defects in waveguide machining on our phase controllers. Since the inevitable defects in waveguide will affect the propagation constant of the waveguide, the discussion of the defects

can be provided by discussing the transmission of the optical field in the waveguide under different propagation constant. Here are details.

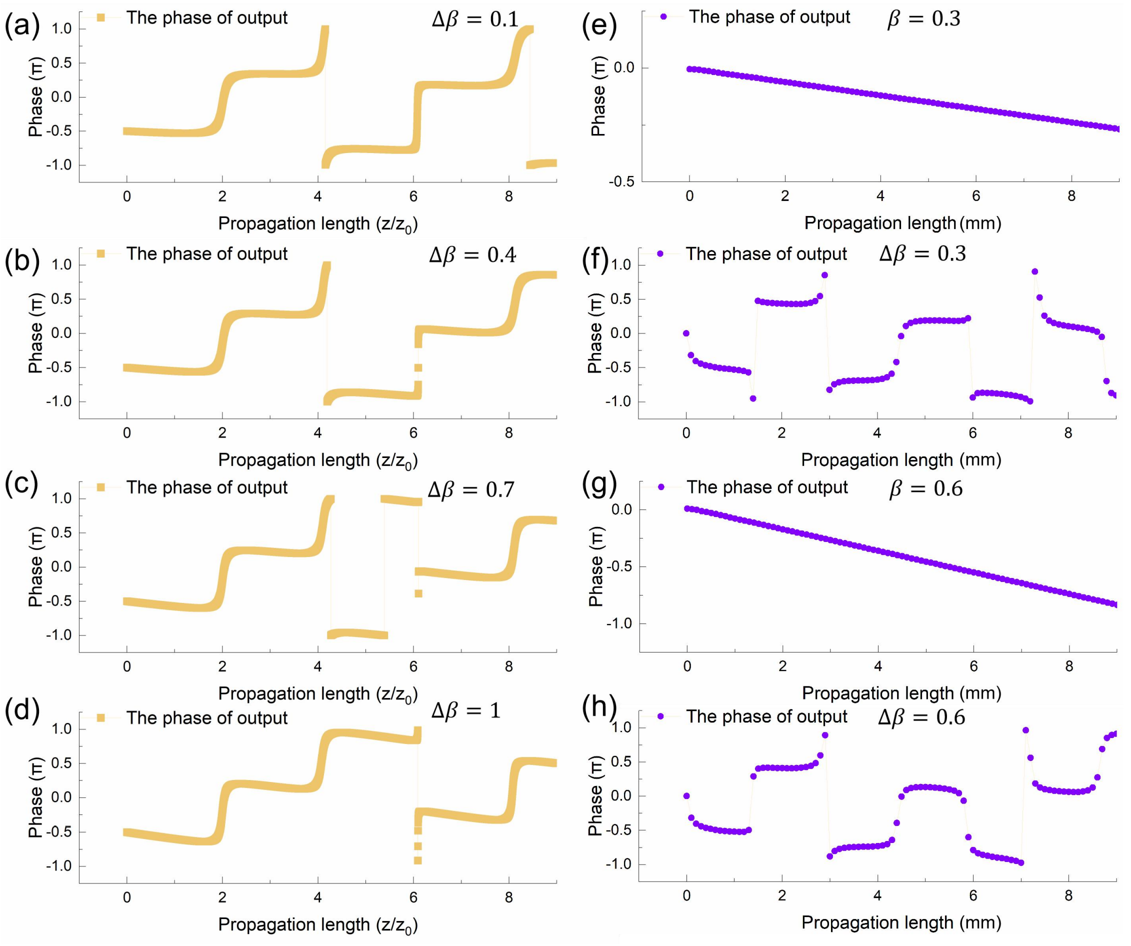

Figure S8. The calculation and simulation to illustrate the impact of inevitable defects in waveguide machining. (a)-(d) The calculation to waveguides with different propagation constant. (e) The simulation to the single waveguide with propagation constant $\beta = 0.3$. (f) The simulation to the phase controller with propagation constant difference $\Delta\beta = 0.3$. (g) The simulation to the single waveguide with propagation constant $\beta = 0.6$. (h) The simulation to the phase controller with propagation constant difference $\Delta\beta = 0.6$.

The Figure S8(a)-(d) shows the calculation to waveguides with different propagation constant. Since our phase controller is a dual-arm interferometer, we are concerned about the difference in propagation constants between waveguides due to defects. In supplementary information III, we proved that the waveguide propagation constant affects the slope of the phase platform. In experiments, the difference of propagation constants between waveguides caused by inevitable defects is often a small amount about 10^{-3}. In the calculation, we consider that the waveguide propagation constant is of the same order as the coupling coefficient. We calculated the different phase changes caused by varying propagation constant β when $\kappa_1 = 1$

and $\alpha = 3$. It can be found that when β is less than 0.4, the phase platform basically does not change (the phase change inside the platform is about 0.01π), while when beta is close to 1, the phase change inside the platform is about 0.07π. This shows that when the perturbation of the propagation constant is within a certain range, the change of the plateau period of the system is small and can be ignored. We further illustrate this with simulations as shown as Figure S8(e)-(h). We simulate a two-arm interferometer consisting of a three-waveguide phase controller and a single waveguide, and a phase interferometer consisting of two independent single waveguides, respectively, analyzing the phase changes caused by the difference of propagation constants in the three-waveguide phase controller and the single waveguide. It is can be found that the phase plateau slope change caused by the difference of propagation constant in a three-waveguide phase controller is much smaller than that caused by the same propagation constant in a single waveguide. This shows that the three-waveguide phase controller is more resistant to the influence of fabrication errors than the single waveguide. In addition, the perturbations of the propagation constant also have practical significance in the experimental process. When all three waveguides are embedded in the same refractive index environment (i.e., silicon oxide), their propagation constants are identical. However, exposing the upper waveguide to air alters its surrounding medium, leading to a propagation constant that differs from those of the lower waveguides. This introduces an effective perturbation to the three-waveguide system. We demonstrate that perturbations in the propagation constant affect the slope of the phase platform in the previous discussion. Therefore, if the upper waveguide is not buried, the resulting variation in propagation constants will cause the phase platform to tilt. In contrast, when the upper waveguide is buried, the phase platform remains level and well-defined in experiments.

Below, we perform a more specific analysis of the phase controller's tolerance for preparation errors. In the laser direct writing process, the radius parameter of the waveguide is possible may have some fabrication error. The laser direct writing fabrication error of the waveguide radius is generally 20-30 nm, and no more than 50 nm. Therefore, the different waveguide radius is also simulated to verify the resistance to fabrication errors of the three-waveguide phase controller. The Figure S9(a) the effect of fabrication error of the waveguide radius.

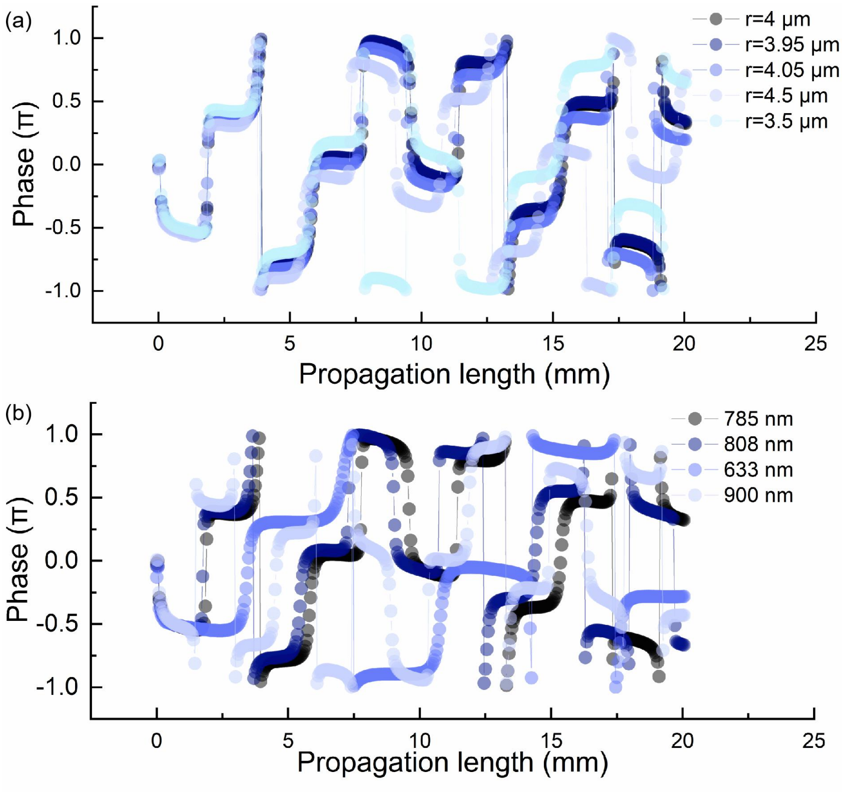

Figure S9. The simulation results about the resistance to fabrication errors and the bandwidth of the three-waveguide configurations based on the laser direct writing platform. (a) The simulation results about the resistance to fabrication errors of the three-waveguide configurations based on the laser direct writing platform. (b) The simulation results about the bandwidth of the three-waveguide configurations based on the laser direct writing platform.

The above simulation results show that the three-waveguide configurations have almost the same secondary full transmission plateau and phase controlling ability with different waveguide radius. At present, the maximum fabrication error of waveguides fabricate by the laser direct writing platform is about 50 nm. If the waveguide with 4 μm radius is taken as the reference, the three-waveguide configurations phase controlling capability and plateau period with the waveguide radius of 3.95 μm and 4.05 μm are almost identical to those of the three-waveguide configurations with the 4 μm waveguide radius. This proves that the waveguide phase controlling capability and plateau period are almost completely unaffected by the fabrication error of the waveguide radius, even under the maximum error 50 nm of the current laser direct writing waveguide machining. However, when the fabrication error reaches 500 nm (Such a large radius offset can hardly be called an “error”), the waveguide phase controlling value of the secondary full transmission plateau changes, but the plateau is still almost identical. This further shows that the three-waveguide phase controller has strong

resistance to fabrication errors.

In addition, we further analyzed the bandwidth of the three-waveguide phase controller fabricated through simulation. The simulation results are shown in the Figure S9(b). Four different bands of light are chosen to simulate the same three-waveguide phase controller. The phase controller operates at the wavelength of 785 nm. When a light with a wavelength similar to 785 nm enters the phase controller, the phase controller can still work. The Figure S9(b) shows that the phase controller has the similar response to the 808 nm light and 785 nm light. When the wavelength of the incident light deviates far from the operating wavelength of the phase controller, it is difficult for the phase controller to have a perfect response. Therefore, the ability of the phase controller to controlling the 633 nm light or the 900 nm is completely different from that of the operating wavelength 785 nm. However, the characteristics of the phase plateau region will still appear with the change of wavelength. This shows that the phase controlling capacity of the controller is different for the light with large deviation from the operating wavelength, but the characteristics of the plateau period will still be retained. The further elaboration is given in supplementary information VI.

In our experiments, in order to evaluate the quality of our waveguides machined, we have collected the CCD images that the signal light transport in the waveguide, showing the uniform scattering light from the waveguide. It verifies that our waveguides are well fabricated and no scattering centers capable of dephasing the guided modes. We present a straight waveguide profile under optical microscope as shown as Figure S10(a). The mode field of the straight waveguide and the mode field of the three-waveguide configurations are shown as Figure S10(b)(c), respectively. This shows that our waveguides are well machined.

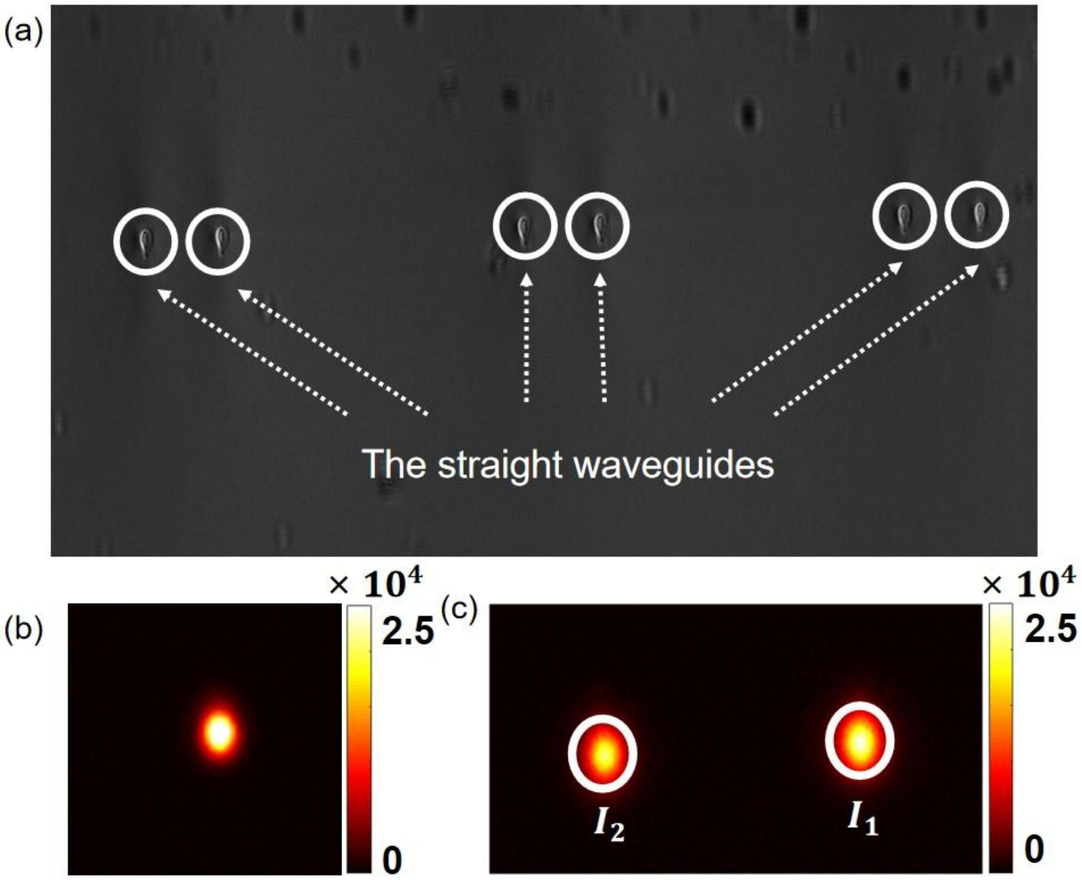

Figure S10. The comparison of mode field between the straight waveguide and the three-waveguide configurations system shown in experiment. (a) The straight waveguide profile. Inside each circle is a straight waveguide. (b) The mode field of the straight waveguide and there is almost no scattering. (c) The mode field of the three-waveguide configurations. The mode field in the three-waveguide configurations is similar to that in the straight waveguide. It verifies that our waveguides are well fabricated and no scattering centers capable of dephasing the guided modes.

In our work, we constructed an on-chip optical permutation circuit based on the on-chip arbitrary phase controller resisting the fabrication error. In order to prove that the third extra waveguide introduced in the permutation circuit mainly realizes phase controlling, a set of supplementary experiments were carried out. Figure S11. shows the experimental results of the permutation circuit without the third extra waveguide ξ and the results of another permutation structure with a third waveguide ξ. It is can be found that the three waveguide systems with different structural parameters d do not affect the function of the on-chip optical permutation circuit. For three cases such as $d = 18$ μm, $d = 19$ μm and $d = 20$ μm, the permutation operation is not affected, and the three-waveguide system has attached an additional phase to it in this case.

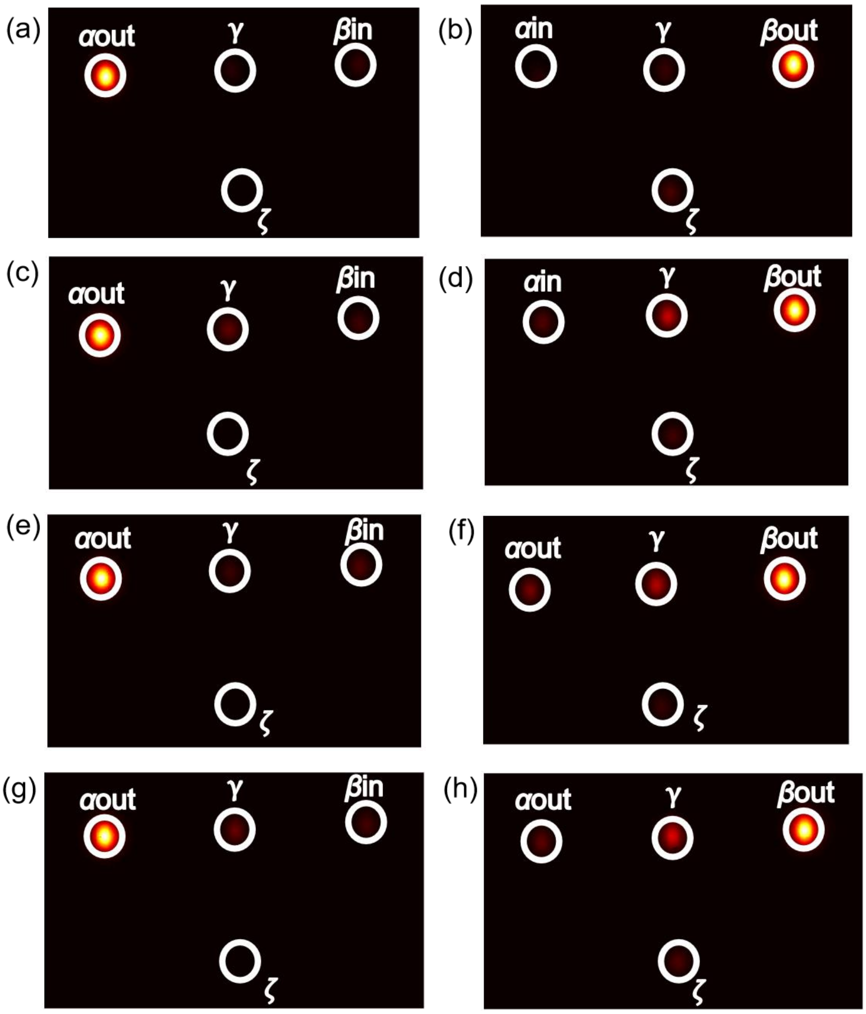

Figure S11. (a)(b) the experimental results of the permutation circuit without the third extra waveguide ξ; (c)(d) the experimental results of the permutation circuit with the third extra waveguide ξ when the geometrical parameter $d = 18$ μm; (e)(f) the experimental results of the permutation circuit with the third extra waveguide ξ when the geometrical parameter $d = 19$ μm; (g)(h) the experimental results of the permutation circuit with the third extra waveguide ξ when the geometrical parameter $d = 20$ μm.

VI. The extra result and verification of the stability of the on-chip silicon-based three-waveguide configurations.

In our work, the three-waveguide phase controller can not only be used as a phase controller based on the laser direct writing platform, but also on the on-chip silicon-based system. Some simulations and experiments based on the on-chip silicon-based system are supplemented to show the excellent phase controlling capability of the three-waveguide configuration.

We design and fabricate the on-chip silicon phase controller based on three-waveguide configurations (as shown in Figure 5 in the main text). The FDTD simulation results of the phase controlling are shown in Figure S12. When the interval between the upper and lower

layers d=0.2 μm, the simulation results show that the phase controlling of 1.39π and 0.92π can be achieved in the second and third light fully transmission regions, respectively. When the interval between the upper and lower layers d=0.25 μm, the simulation results show that the phase controlling of 0.26π and 1.66π can be achieved in the second and third light fully transmission regions, respectively. The simulation results also show that the phase controlling of 1.925π and 1.215π can be achieved in the second and third light fully transmission regions, respectively when d=0.35 μm. The phase controlling of 1.87π and 1.16π can be achieved in the second and third full transparent regions, respectively, when d=0.4 μm. The results of 0-2π can be covered.

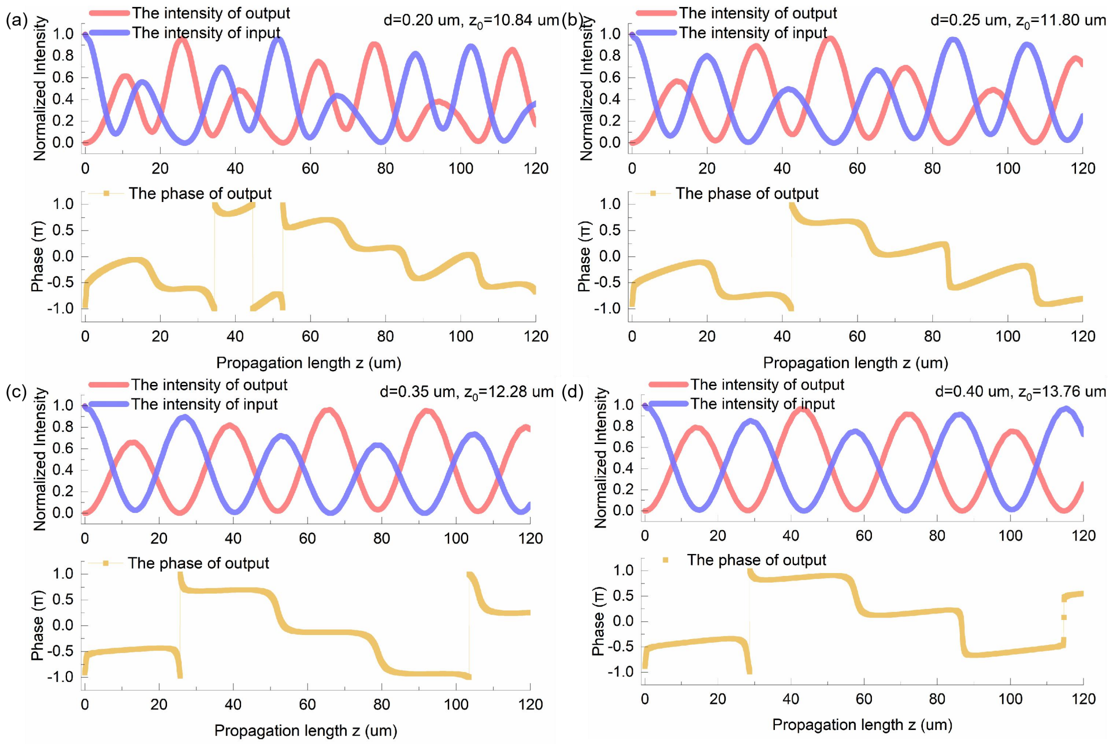

Figure S12. The results of FDTD simulation of the on-chip silicon-based three-waveguide configurations. (a) The phase distributions in waveguide C of the three-waveguide configuration when d=0.2 μm, the length of the plateau period is about 10 μm; (b) The phase distributions in waveguide C of the three-waveguide configuration when d=0.25 μm, the length of the plateau period is about 15 μm; (c) The phase distributions in waveguide C of the three-waveguide configuration when d=0.35 μm, the length of the plateau period is about 20 μm;(d) The phase distributions in waveguide C of the three-waveguide configuration when d=0.4 μm, the length of the plateau period is about 20 μm.

When the thickness of silicon oxide layer *d* is different, the plateau length of phase controller is different, which is proportional to the complete coupling length z_0 (z_0 is the length of the waveguide when the light first reaches the highest transmission in the three-waveguide configuration). These simulation results further prove that our three-waveguide phase

controller has excellent resistance to fabrication errors.

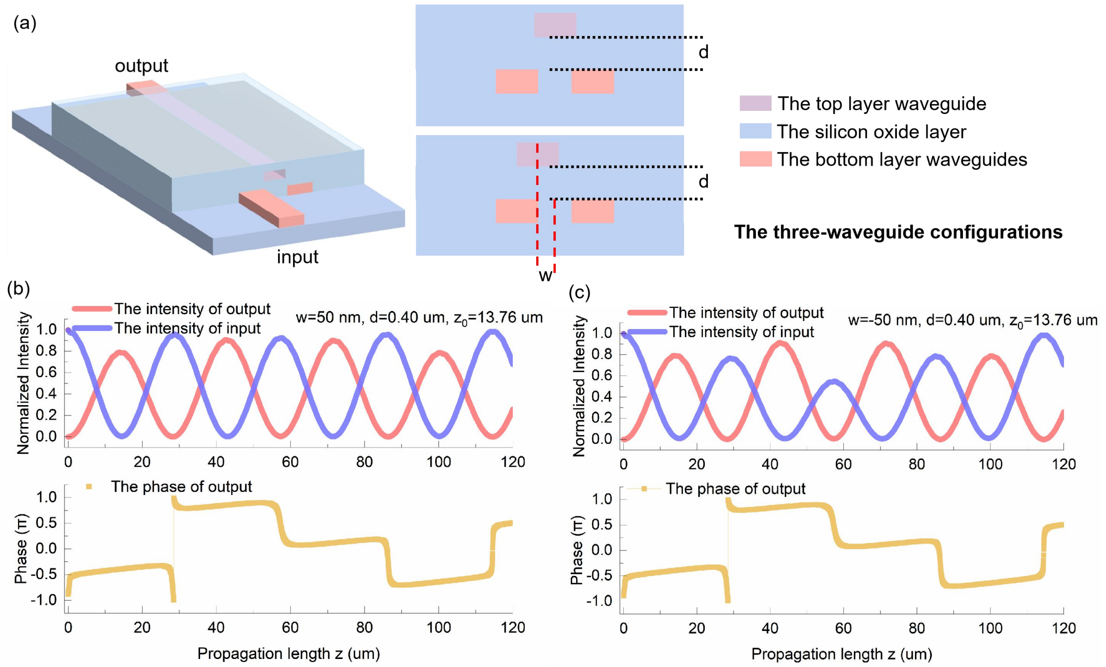

Figure S13. The simulation verification of the robustness of the on-chip silicon-based three-waveguide configurations. (a) The fabrication error *w* between the top layer and the bottom layer waveguides; (b) The simulation result in the output waveguide of the three-waveguide configuration when w=50 nm; (c) The simulation result in the output waveguide of the three-waveguide configuration when w=-50 nm.

On this basis, we further study the resistance of the three-waveguide configuration to fabrication error. Whether on the laser direct writing platform or on the on-chip silicon-based system, it is very easy to manufacture perfectly identical waveguides, which have small propagation loss. Therefore, the most important fabrication error for an on-chip silicon-based three-waveguide configuration is not the parameters of the single waveguide itself, but the relative positions of the top layer and the bottom layer waveguides. It is difficult for the top layer waveguide to accurately align the center of the bottom layer two waveguides experimentally. The Figure S13(a) shows the fabrication error *w* between the top layer and the bottom layer waveguides. The fabrication error is about 20-30 nm in the fabrication process. The fabrication error of 50 nm was selected for simulations and experiments, which is much larger than the actual fabrication error 20-30 nm. The FDTD simulation result is shown in Figure S13(b)(c) when the thickness of the silicon oxide layer d=400 nm. Whether w=50 nm (50 nm to the right) or w=-50 nm (50 nm to the left), the FDTD simulation result is almost exactly same as that when w=0 as shown in Figure S12(d). We further demonstrated this through experiments. The Detailed results can be found in Table S3:

Table S3. The simulation verification of the robustness of the on-chip silicon-based three-waveguide configurations.

d/μm	Theory ϕ (*π)	Experimental ϕ (*π)		
		w=0 nm	w=-50 nm	w=50 nm
0.2	-0.7	-0.68	-0.67	-0.79
0.25	-0.75	-0.67	-0.70	-0.63
0.4	0.80	0.82	0.84	0.79

We measured the accumulated phase generated by phase controllers with different *w*, finding minimal phase differences between them. This demonstrates the resistance to the fabrication error *w* between the top layer and the bottom layer waveguides.

In addition, we further prove that the on-chip silicon-based phase controller has a very wide bandwidth through simulation. The simulation results are shown in the Figure S14.

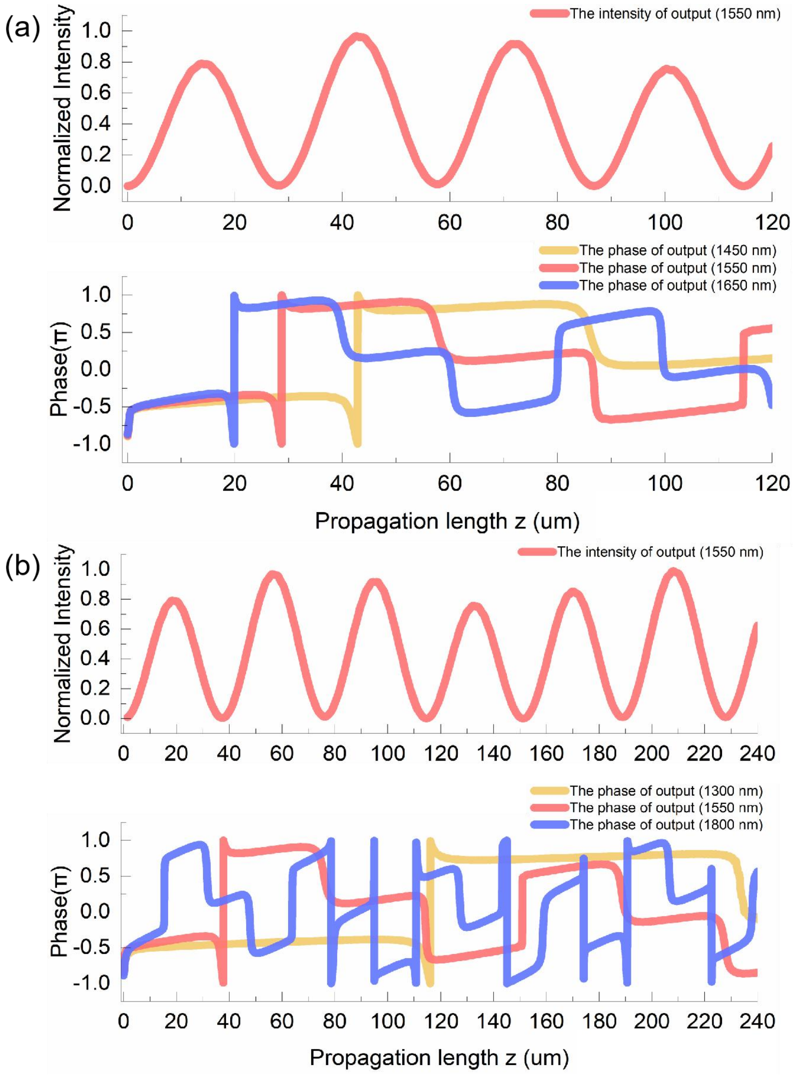

Figure S14. The simulation results of the bandwidth of the on-chip silicon-based three-waveguide configurations. (a) The simulation results of the phase controller with the wavelength range from 1450 nm to 1650 nm; (b) The simulation results of the phase controller with the wavelength range from 1300 nm to 1800 nm.

In the wavelength range from 1450 nm to 1650 nm, the phase controlling plateau period is partially overlaps for the different wavelengths of light, and the phase value is almost equal in the second fully transmittance region. This shows that when the central wavelength of the incident light is 1550 nm, the phase controller can play the same controlling role for the light within the 200 nm bandwidth. However, in the wavelength range from 1300 nm to 1800 nm, the phase controlling value of the phase controller is also almost equal to the light with the three different wavelengths of 1300 nm, 1550 nm, 1800 nm, but the phase controlling plateau period is no longer overlapping. The phase controller no longer acts on all light in this wavelength range. Therefore, the bandwidth of the phase controller is at least 200 nm. Further, the phase controlling capability of the three-waveguide phase controller is the same for all wavelengths of light for the same plateau, and the phase controlling value is not affected by the change of wavelength. However, the position of the plateau region will change with the change of wavelength, which will affect the bandwidth of the device. Due to the long plateau period of the three-waveguide phase controller, the controller has a wide bandwidth, which further explains the advantages of the three-waveguide phase controller.

VII. The schematic of the optical measurement setup.

The schematic diagrams for femtosecond laser direct writing measurement and on-chip measurement are as shown as Figure S15.

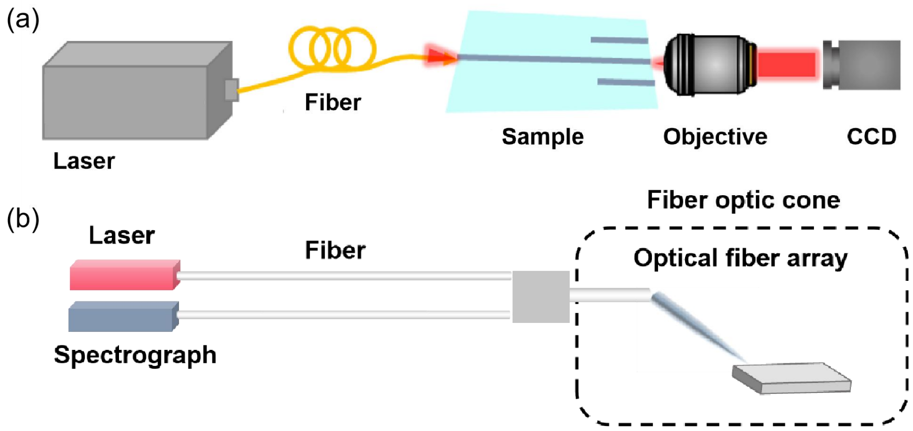

Figure S15. the schematic of the optical measurement setup. (a) the optical measurement setup based on the femtosecond laser direct writing platform; (b) the optical measurement setup based on the on-chip silicon

photonic chip platform.

For the femtosecond laser direct writing platform, we adopt the optical measurement setup as shown in Figure S15 (a). The light emitted from the laser passes through the fiber and enters the sample. Then the light passing through the sample is collected by the objective lens and finally enters the CCD.

For the silicon-based on-chip platform, we adopt the optical measurement setup as shown in Figure S15 (b). The light emitted from the laser passes through the fiber and enters the optical fiber cone platform. After the signal light is coupled into the sample through the transmitting port of the optical fiber array, the light passing through the sample exits from the collecting port of the optical fiber array and finally enters the spectrometer.
